# Comprehensive investigation on the synergistic antibacterial activities of *Jatropha curcas* pressed cake and seed oil in combination with antibiotics

**DOI:** 10.1186/s13568-019-0793-6

**Published:** 2019-05-17

**Authors:** Abdul Haq, Maleeha Siddiqi, Syeda Zakia Batool, Arshad Islam, Alam Khan, Dildar Khan, Samiullah Khan, Haji Khan, Aamer Ali Shah, Fariha Hasan, Safia Ahmed, Malik Badshah

**Affiliations:** 1 Department of Microbiology, Faculty of Biological Sciences, Quaid-i-Azam University, Islamabad, 45320 Pakistan; 2 Postgraduate Program in Physiology and Pharmacology, Institute of Biological Sciences, Universidade Federal de Minas Gerais, Belo Horizonte, Minas Gerais 31270-901 Brazil; 3 Department of Pharmacy, Quaid-i-Azam University, Islamabad, 45320 Pakistan; 4 Centre for Biotechnology and Microbiology, University of Swat, Swat, 19200 Pakistan; 5 Present Address: Sulaiman Bin Abdullah Aba Al Khail Centre for Interdisciplinary Research in Basic Sciences, International Islamic University, Islamabad, 44000 Pakistan

**Keywords:** *Jatropha curcas*, Antibiotics, Multidrug resistant bacteria, Combinatorial therapy, Fractional inhibitory concentration index, Synergism

## Abstract

Synergistic combinations of various antimicrobial agents are considered ideal strategies in combating clinical and multidrug resistant (MDR) infections. In this study, antibacterial potential of *Jatropha curcas* crude seed extracts, seed oil, commercially available antibiotics, and their combinations were investigated for their synergistic effect against clinical, MDR and ATCC bacterial strains by agar well diffusion assay. Methanolic extracts remained more active against *Staphylococcus aureus* (ATCC), with zone of inhibition (ZOI) of 21 mm, than clinical and methicillin-resistant *S. aureus* (MRSA) strains (ZOI range ~ 15.0–17.0 mm). Molecular docking demonstrated that beta-monolaurin from methanolic extract exhibited greater affinity conformation for UDP-*N*-acetylmuramoyl-tripeptide–d-alanyl-d-alanine (MurF) ligase’s active pocket with binding energy of -7.3 kcal/mol. Moxifloxacin exhibited greater activity against *Escherichia coli* (ATCC) (ZOI ~ 50.0 mm), followed by ofloxacin against *Pseudomonas chlororaphis* (47.3 mm), moxifloxacin against *P. monteilii* (47 mm)*, P. aeruginosa* (46.3 mm) and MRSA2 (46 mm) and ofloxacin against *S. aureus* (ATCC) strains (45.7 mm). Methanolic extract in combination with rifampicin showed the highest synergism against MRSA strains, *A. baumannii*, *E. coli, E. faecalis, S. aureus,* and *P. aeruginosa*, *A. baumannii* (MDR strain), *P. chlororaphis*, *E. coli* ATCC25922 and *S. aureus* ATCC25923. In combinations, moxifloxacin exhibited the highest antagonism. The methanolic, *n*-hexane, aqueous extracts and seed oil in various combinations with antibiotics showed 44.71, 32.94, 9.41 and 25.88% synergism, respectively. The current study showed that potency of antibiotics was improved when screened in combination with *J. curcas* seed’s components, supporting the drug combination strategy to combat antibacterial resistance.

**Electronic supplementary material:**

The online version of this article (10.1186/s13568-019-0793-6) contains supplementary material, which is available to authorized users.

## Introduction

Over the last few decades, the exhaustive over-prescription, and self-medication of clinically available antibiotics and consequently the long-term exposure of pathogenic microorganisms to these antibiotics has led to the development of antibiotic resistance (Harbottle et al. 2006). The mechanism behind the antibiotic resistance, as a result of long-term exposure, is the accumulation of multiple genes, each coding for resistance to a single drug. This mechanism within a single bacterial cell has aided in the emergence of multidrug resistant (MDR) bacteria. MDR bacteria use horizontal gene transfer to spread the antibiotic resistance genes among themselves (Odonkor and Addo 2011). A number of diseases caused by MDR bacterial strains are incurable and fatal due to their high resistance rate towards most of the clinically available antibiotics (Nikaido 2009). Currently, more than 70% of the pathogenic bacteria are reported to have acquired resistance against antibiotic therapies (Harvey et al. 2015). In this context, the development of novel, efficacious, cost-effective and non-cross resistant antibiotics has become the only alternative to treat bacterial infection and remains a great challenge for pharmaceutical industries in terms of exploring novel and effective drugs as well as drug development expenditure (Sharma et al. 2016).

Historically, medicinal plants or their extracts have been used as traditional medicine to treat various infectious. Numerous plants or their extracts have been reported to possess antimicrobial properties (Rachana et al. 2012). Plants or their products may act as bactericidal as well as bacteriostatic agents against microbial efflux pump, quorum sensing and biofilm formation (Savoia 2012). During the last few decades, investigations on the antimicrobial potential of natural products remained a great focus for drug discovery around the world (Rios and Recio 2005). However, only a few medicinal plants or their extracts were managed to reach clinical trials. As of now, not even a single antimicrobial agent derived from medicinal plants has been officially approved. Different technical challenges have been sought and possible recommendations have been proposed for development of drugs derived from natural compounds (Cos et al. 2006). Mixtures of commercially available pharmaceutical and herbal remedies against different ailments have been reported for traditional use in self-medication (Buchness 1998; Donaldson 1998). Plant remedies used in combination with pharmaceutical drugs have certain herb-to-drug interactions and the possible outcomes of these interactions include synergistic amplification of the antimicrobial potential and reduction in the adverse side effects of synthetic drugs. These combined effects have certainly reduced the chances of lower efficacy of drugs used alone to treat a microbial infection for a long time (Borchers et al. 1997). Based on the traditional herb-to-drug combination strategy, the ineffective synthetic antibiotics at present can be used in combination with the inexpensive, handy and harmless medicinal plants. The herb-to-drug combination strategy may lead to the discovery of novel antibiotics and the re-use of those antibiotics towards which bacteria have developed resistance (Saklani and Kutty 2008).

*Jatropha curcas* is a multipurpose shrub belonging to *Euphorbiaceae* family and its seeds contain oil that can be used for biodiesel production and assayed for antimicrobial potential as well. It can sustain itself in sub-tropical, semi-arid, saline and acidic soil regions. Traditionally, it has a long history of medicinal use and has been greatly utilized in treating bacterial as well as fungal infections. Various extracts of *J. curcas* were phytochemically analyzed and reported to have antimicrobial activities against different human pathogens (Ajayi 2018; Arekemase et al. 2011). However, only a few reports are available on antimicrobial activities of pressed cake (de-oiled seed) of *J. curcas* and that is mostly restricted to standard cultures such as American type culture and collection (ATCC) strains.

In the present study, *J. curcas* de-oiled seed cake and seed oil were investigated for their phytochemical constituents analysis and antibacterial potential against clinical bacterial pathogenic isolates, MDR and ATCC bacterial strains. Moreover, for the first time, the fractional inhibitory concentrations (FIC) of various extracts of de-oiled seed cake and seed oil of *J. curcas* in combination with the various commercially available antibiotics against selected bacterial strains have been studied in order to investigate their synergistic, antagonistic, indifferent and additive effects.

## Materials and methods

### *Jatropha curcas* seed oil extraction

The local variety of *J. curcas’* seeds was obtained from local dealer and identified at the Department of Plant Sciences, Quaid-i-Azam University, Islamabad. Oil was extracted from whole seeds of *J. curcas* plant using mechanical oil expeller. After extracting oil, the de-oiled seed cake was preserved in sterile zipper bags at 4 °C and the oil was stored in the dark for further use.

### Preparation of extracts, commercial antibiotics’ solutions and their combinations

De-oiled seed extracts of *J. curcas* plant were prepared as previously described (Basri and Fan 2005). 100 g of fine powdered de-oiled seed cake of *J. curcas* was dissolved in 500 mL of water, methanol or *n*-hexane and incubated at 30 °C for 48 h, in a shaking incubator at 100 rpm. The solution was filtered through Whatman filter paper and the filtrate was concentrated at 45 °C under reduced pressure in rotary evaporator. The concentrated filtrate was allowed to dry at room temperature. The yield of methanolic, *n*-hexane and aqueous extracts were 17.0, 10.39 and 9.04%, respectively. Each extract (200 mg) was dissolved in 1 mL dimethyl sulfoxide (DMSO) for further use. Stock solutions of the selected commercially available antibiotics (powder form) including ciprofloxacin (Global Pharmaceuticals), cefotaxime (Global Pharmaceuticals), rifampicin (Pfizer Laboratories Limited) and moxifloxacin (Bio Labs Pak (Pvt) Limited) were prepared at a concentration of 100 µg/mL in de-ionized water. Ofloxacin (GlaxoSmithKline Pakistan Limited), the broad spectrum antibiotic that is active against various Gram positive and Gram negative bacteria, was used as a positive control. The *J. curcas* seed oil, de-oiled seed extracts and the antibiotics were filtered using sterile syringe filter (0.2 µm pore size). Commercially available antibiotics were used in combination with *J. curcas* seed oil and its de-oiled seed cake extracts. For combinatorial activities, each extract and antibiotic solution was taken in 1:1 volume in sterile tubes. 100 µL of seed oil, each extract and antibiotic were individually as well as in combinations spread on Mueller–Hinton agar (MHA) and the plates were incubated at 37 °C for 24 h to confirm sterility.

### Preliminary phytochemical screening

The preliminary qualitative phytochemical screening of *J. curcas* seed oil and de-oiled seed cake was carried out for identification of balsams, flavonoids, saponins, glycosides, saponin glycosides, steroids, volatile oils and tannins by methods previously reported (Amina et al. 2013; Arekemase et al. 2011; Sajjad et al. 2015).

### *Characterization* of *J. curcas seed oil and de*-*oiled seed* extracts

The *J. curcas* seed oil and seed extracts were analyzed by FTIR (Bruker Tensor 27) absorption spectra registered for *J. curcas* seed oil and de-oiled seed extracts in the range of 4000–400 cm^−1^.

The chemical composition of *J. curcas* seed oil and de-oiled seed cake extracts (aqueous, methanolic and *n*-hexane extracts) was also analyzed by gas chromatography coupled with mass spectrometry (GC–MS) technique (GC–MS—QP5050A, Shimadzu, Europe) according to the previously described methods (Mu’azu et al. 2013; Oskoueian et al. 2011) with some modifications as discussed below. For GC–MS analysis of *J. curcas* seed oil and de-oiled seed cake extracts, some of the conditions were varying and then a 2 µL of each sample (12.5 mg/mL) was injected in column using automated injector with a split ratio of 1/48 and 1/25 for *J. curcas* de-oiled seed cake extracts and seed oil, respectively. A DB-5 column was used with a length of 30 m, internal diameter of 0.25 mm and thickness of 0.25 μm, while flow rate was maintained at 1 mL/min and 1.8 mL/min for de-oiled seed cake extracts (methanolic, *n*-hexane and aqueous) and seed oil, respectively. Thermal conductivity detector was used for detection of analytes. The identification of the peak was based on computer matching of mass spectra with National Institute of Standards and Technology library. The mass to charge scanning ranged from 40 to 600 amu.

### Collection and maintenance of bacterial cultures

Three types of bacterial strains including commonly occurring Gram positive and Gram negative human pathogenic clinical isolates (*Acinetobacter baumannii, Escherichia coli, Proteus vulgaris, Pseudomonas aeruginosa, Enterococcus faecalis and Staphylococcus aureus*), MDR Gram positive and Gram negative bacterial strains [*Pseudomonas monteilii*, *Pseudomonas chlororaphis, Klebsiella pneumoniae*, *Acinetobacter baumannii* (MDR), and methicillin-resistant *Staphylococcus aureus* (MRSA) strains (MRSA1, MRSA2, MRSA3, MSSA4 and MRSA5)] were selected. These strains were selected because they are considered most challenging in terms of antibiotic susceptibility and cause various infections in a large population. All the strains were obtained from Pakistan Institute of Medical Sciences, Islamabad. In addition, the two ATCC strains, *Escherichia coli* (ATCC 25922) and *Staphylococcus aureus* (ATCC 25923) were used as reference strains (positive controls). Each strain was grown and maintained on nutrient agar media at 4 °C and sub-cultured on fresh media at regular intervals. The antibiotic resistance profiling of MDR strains was confirmed by disc diffusion method (see Additional file 1: Table S1).

### Preparation of bacterial culture for antibacterial assay

Bacterial cultures were prepared for antibacterial assay according to the method of Gahlaut and Chhillar (2013). The bacterial strains, under aseptic conditions, were incubated and grown in nutrient broth at 37 °C for 24 h and centrifuged at 4000 rpm for 5 min. Supernatants were discarded and pellets were re-suspended in 20 mL sterilized normal saline, followed by centrifugation at 4000 rpm for 5 min. The pellet obtained was suspended in sterile normal saline, labeled accordingly and its optical density (OD) was measured at 600 nm wavelength using ultraviolet visible spectrophotometer (8453 UV–Visible spectrophotometer). The bacterial suspension was diluted with normal saline until the OD was in range of 0.5–1.0 that corresponds to 5 × 10^6^ CFU/mL (Sarker et al. 2007).

### Antibacterial assay

Antibacterial potential of *J. curcas* seed oil, de-oiled seed extracts and the selected commercially available antibiotics was evaluated using agar well diffusion method of Boakye-Yiadom and co-workers (1979). The standardized inocula (5 × 10^6^ CFU/mL) were swabbed onto respective plates containing MHA growth media using sterile cotton swabs. A sterile copper borer of 8 mm diameter was used to create wells in the solidified growth medium in the plates. Each well was properly labeled and filled with 100 µL of *J. curcas* seed oil, de-oiled seed extracts and the selected commercially available antibiotics, independently. Ofloxacin was used as a positive control. DMSO (99.9%) and de-ionized water was used as negative control for seed extracts and antibiotics. The inoculated petri plates were left for an hour at room temperature to allow for diffusion of treatments before the bacterial growth commenced. The plates were then incubated at 37 °C for 24 h, followed by the measurement of zones of inhibition (ZOI) around the wells.

### Molecular docking with AutoDock

An AutoDock 4 on Intel was used for molecular docking of chemical compounds detected in methanolic residues by GC–MS analysis. The docking was carried out into the active sites of *Acinetobacter baumannii* UDP-*N*-acetylmuramoyl-tripeptide–d-alanyl-d-alanine (MurF) (MurF) receptor (PDB, ID, 4QDI). A 32 bit operating system Intel Core™ i5 CPU M 540 @ 2.53 GHz was used and in order to cover the active sites, the grid was set manually and centrally along the X, Y and Z axis as − 29.60, 2.43 and − 2.59 with 10 Å dimensions accordingly. All other parameters were kept at default.

### Minimum inhibitory concentration

Minimum inhibitory concentrations (MIC) of all treatments (*J. curcas* seed oil, de-oiled seed extracts and antibiotics) were determined using agar well diffusion method. The said treatments were diluted by twofold dilutions. The seed oil, de-oiled seed extracts and antibiotics dilutions prepared were in the range of 0.097–100 mg/mL and 0.049–100 µg/mL, respectively. A standardized inoculum (5 × 10^6^ CFU/mL) of each target bacterial strain was swabbed on the MHA plate, a 100 µL of each treatment was added in the respective well and incubated overnight at 37 °C for 24 h. The lowest dilution of each treatment that gave a clear inhibition zone was considered as MIC of the respective treatment. Sterility of solutions was maintained throughout the experiments.

### Synergistic antibacterial assay

Synergistic antibacterial potential of each *J. curcas* de-oiled seed extract and its seed oil in combination with ofloxacin, ciprofloxacin, moxifloxacin, cefotaxime and rifampicin was determined against selected bacterial strains by checkerboard method as previously described (Farooqui et al. 2015) with some modifications. The MHA plates were swabbed with standardized inoculum (5 × 10^6^ CFU/mL) followed by the addition of 100 µL of various serial dilutions of the combination of respective drugs (mixture of 50 µL of *J. curcas* seed oil or de-oiled seed extract and 50 µL of selected antibiotic) in respective wells. The plates were left at room temperature for an hour in order to allow the combined drugs to properly diffuse in media before incubation at 37 °C for 24 h. The final seed extract or antibiotic concentration for clinical, MDR and reference strains was about 0.097 to 200 mg/mL or 0.049 to 100 µg/mL, respectively. Fractional inhibitory concentration index (FICI) for each combination was determined using Eq. 1.

1 $$\sum {\text{FICI }} = {\text{FIC }}\left[ {\text{A}} \right] + {\text{FIC }}\left[ {\text{B}} \right]$$

where [A] = *J. curcas* seed oil or de-oiled seed extracts; [B] = antibiotics; FIC [A] = MIC of agent A in combination/MIC of agent A alone and FIC [B] = MIC of agent B in combination/MIC of agent B alone.

The FICI for each combination was determined as described in the literature (Hossain et al. 2016) and is given as follows: FICI ≤ 0.5 = synergy; FICI > 0.5 or ≤ 1 = additive; and FICI > 1 or ≤ 4 = indifference; and FICI > 4 = antagonism.

### Statistical analyses

All the assays were carried out in triplicate and data was presented as the mean of three independent experiments ± standard deviation (SD). One-way analysis of variance (ANOVA) followed by *Bonferroni’s post*-*test* for multiple comparisons were applied to compare the antimicrobial activities of individual extracts and antibiotics independently as well as in combination using GraphPad Prism Software version 6.0 (La Jolla, CA, USA).

## Results

### Chemical characterization of *J. curcas* seed oil and de-oiled seed extracts

In the present study, a number of conventional phytochemical analyses were carried out to elucidate the composition of *J. curcas* seed oil and de-oiled seed cake. Different qualitative chemical tests were performed to investigate the major phytochemical compounds/groups present in *J. curcas* de-oiled seed cake and seed oil. During conventional phytochemical screening, different phytochemicals such as flavonoids, tannins, saponin glycosides and steroids were found in de-oiled seed cake. Similarly, in seed oil, the phytochemicals such as flavonoids, tannins, saponins, glycosides and steroids were found. In addition, characterization of chemical composition of *J. curcas* pressed cake and seed oil was carried out using different spectroscopic techniques. The analyses of FTIR spectra (see Additional file 1: Figures S1–S4) obtained for *J. curcas* de-oiled seed extracts (methanolic, *n*-hexane and aqueous) also revealed the presence of a broad range of compounds such as ester linkages, amide linkages, carbon–hydrogen bonds, aromatic functional groups and carbonyl linkages that correspond to the presence of carbohydrates, cellulose, hemicellulose and lignin components. The corresponding different functional groups present in all extracts of *J. curcas* extracts are shown in Additional file 1: Tables S2–S5. FTIR spectroscopy is used to identify specific functional groups present in an organic, polymeric, inorganic compound or other material (Chen et al. 2015).

**Fig. 1:**
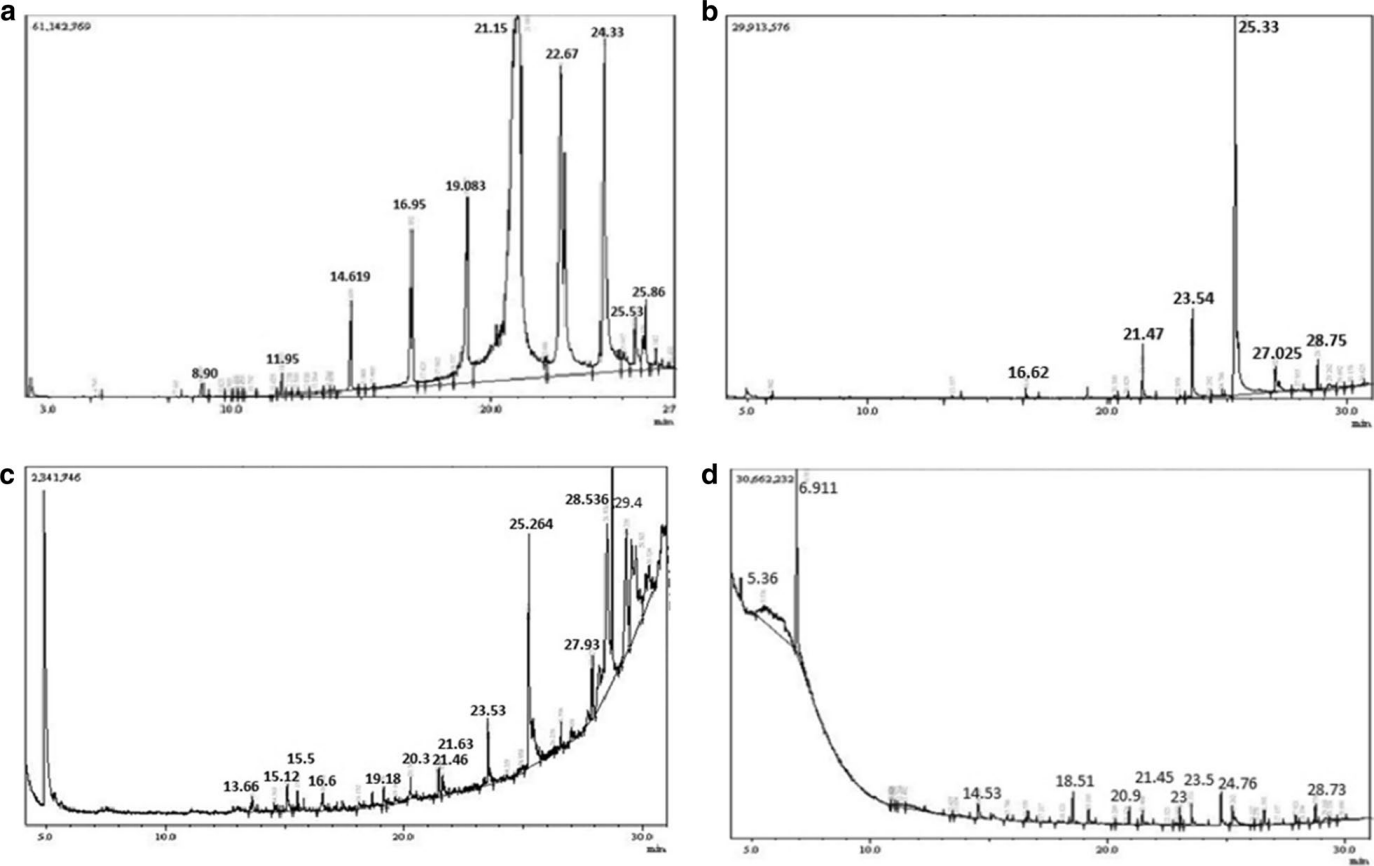
Gas chromatography coupled with mass spectrometry (GC–MS) chromatogram obtained for *J. curcas* de-oiled pressed cake extracts and seed oil, **a** GC–MS chromatogram obtained for *J. curcas* seed oil, **b** GC–MS chromatogram obtained for *n*-hexane extract of *J. curcas* de-oiled seed cake, **c** GC–MS chromatogram obtained for methanolic extract of *J. curcas* de-oiled seed cake, **d** GC–MS chromatogram obtained for aqueous extract of *J. curcas* de-oiled seed cake

Moreover, the GC–MS spectrophotometric analyses of *J. curcas* seed oil and de-oiled seed cake extracts (Fig. 1) showed the presence of a wide range of bioactive compounds. About 16 different saturated and non-saturated long chain fatty acids were determined in *J. curcas* seed oil (see Additional file 1: Table S6). Similarly, in *n*-hexane extract, both saturated and unsaturated fatty acids such as oleic acids, 9,12-octadecadienoic acid (*Z*,*Z*)-, palmitic acid and myristic acid were identified (see Additional file 1: Table S7). Conversely, GC–MS analysis of methanolic extract presented diverse compounds such as I-(+)-ascorbic acid 2,6-dihexadecanoate, 9-hexadecenal, beta-monolaurin, bis(tridecyl) phthalate, 1-docosanol and diacetone alcohol (see Additional file 1: Table S8). The GC–MS analysis of aqueous extract also indicated the presence of different compounds such as 1,4-dithiane, dodecanoic acid methyl ester, methyl tetradecanoate, vitamin D3, methyl ester, palmitic acid, isopropyl linoleate and di-*n*-octyl phthalate (see Additional file 1: Table S9). In addition, the identified structures, areas and heights of the peak are given in Additional file 1: Tables S6–S9.

### Antibacterial assay

In the current study, antibacterial activities of *J. curcas* seed oil, de-oiled seed cake extracts, and commercially available antibiotics (ofloxacin, ciprofloxacin, moxifloxacin, cefotaxime and rifampicin) were used against the selected clinical pathogenic isolates, MDR and standard ATCC bacterial strains. In addition, synergistic activities of all these components in combination with aforementioned antibiotics were also investigated against each selected bacterial strain.

**Fig. 2:**
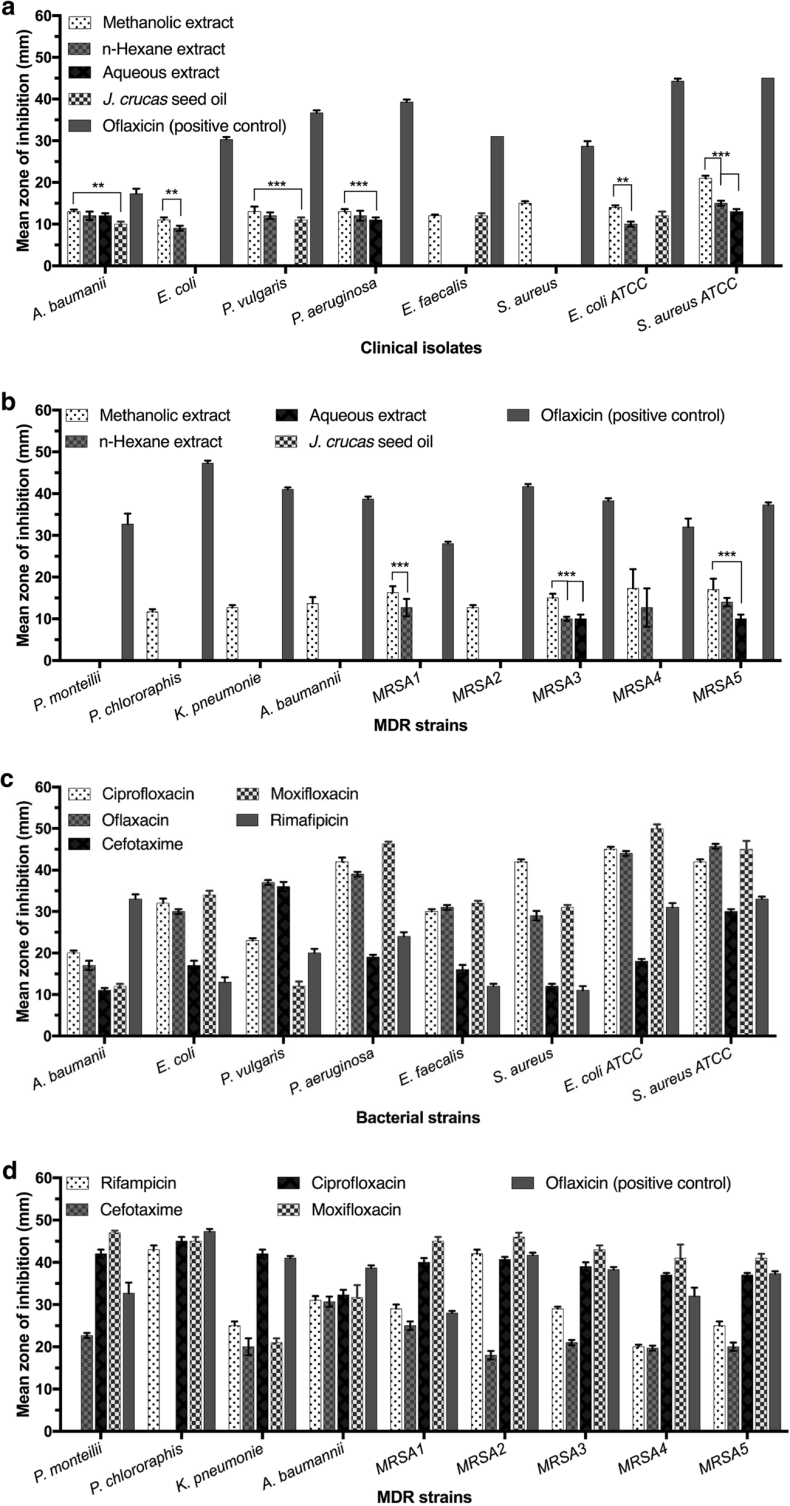
Antibacterial activities of *J. curcas* de-oiled pressed cake extracts (200 mg/mL DMSO), seed oil (200 mg/mL DMSO) and commercially available antibiotics (100 µg/mL in de-ionized water) against clinical, MDR and ATCC bacterial strains. **a** Antibacterial activities of *J. curcas* de-oiled pressed cake extracts and seed oil against clinical and ATCC bacterial strains, **b** antibacterial activities of *J. curcas* seed oil and de-oiled seed cake extracts against selected MDR strains, **c** antibacterial activities of commercially available antibiotics against selected clinical and ATCC bacterial isolates, **d** antibacterial activities of commercially available antibiotics against selected MDR bacterial isolates. 100 µL of solution of each extract, seed oil, antibiotic and positive control (ofloxacin) was added to the respective wells punched in MHA plates, pre-swabbed with respective clinical isolates and incubated at 37 °C for 24 h. All the tests were carried out in triplicate. The mean zones of inhibition (mm) created by the respective treatments against each bacterial strain were recorded. The bigger the mean zone of inhibition (mm), the higher was considered the susceptibility of bacterial strains. Data presented are means of three independent experiments ± SD. *P < 0.05; **P < 0.01, ***P < 0.001 (for comparisons of all treatments by one-way ANOVA, followed by *Bonferroni’s* post test)

When the extracts and seed oil were individually evaluated against clinical isolates, the methanolic extract exhibited significant (*P* < 0.001) antibacterial activity (ZOI 15 mm) against *S. aureus* as compared to those of seed oil, *n*-hexane and aqueous extracts (Fig. 2a). While for *n*-hexane and aqueous extracts or seed oil, the *S. aureus* was found to be the most resistant bacterial strain with no antibacterial activity against it. Among the clinical isolates, *A. baumannii* strain was found to be susceptible to all extracts but the activity of methanolic extract was significantly higher as compared to that of seed oil (*P *< 0.05) as shown in Fig. 2a. The ZOI exhibited by seed oil, aqueous, *n*-hexane and methanolic extracts against *A. baumannii* in (Fig. 2a) were 10, 12, 12 and 13 mm, respectively. Overall, the methanolic extract, against clinical isolates, remained more active than the other de-oiled seed extracts and oil. In addition, for ATCC reference strains, the highest antibacterial activity was exhibited by methanolic extract with ZOI ~ 21 mm against *S. aureus* (ATCC 25923) (Fig. 2a). The antibacterial activity of methanolic extract was more significant (*P *< 0.001) than *n*-hexane, aqueous extract and seed oil against *S. aureus* (ATCC 25923).

Similarly, in case of MDR strains, the highest antibacterial activity was exhibited by methanolic extract (Fig. 2b) with ZOI ~ 17 mm against methicillin resistant *S. aureus* (MRSA4 and MRSA5) strains and was found significantly higher (*P* < 0.001) than that exhibited by seed oil and aqueous extract. The *n*-hexane extract was found second most active extract with ZOI of 13 mm against MRSA5 (Fig. 2b) and was significantly more active (*P *< 0.001) than seed oil. Overall, methanolic extract exhibited the most potent antibacterial activities (Fig. 2b) compared to seed oil, *n*-hexane and aqueous extracts against MDR strains. *J. curcas* seed oil did not exhibit any antibacterial activity against any MDR strain. The most resistant MDR strain was *P. monteilii* against which none of the extracts and seed oil exhibited any antibacterial activity.

In case of antibiotics for clinical isolates, the highest antibacterial activity was exhibited by moxifloxacin with a ZOI ~ 46 mm against *P. aeruginosa* (Fig. 2c) and was significantly higher (*P *< 0.001) than that of ciprofloxacin, cefotaxime, rifampicin and ofloxacin (control drug). Moxifloxacin exhibited comparatively more potent antibacterial activities against all clinical strains except *P. vulgaris* and *A. baumannii* (Fig. 2c). Rifampicin and cefotaxime had the least potent antibacterial activities against all selected clinical isolates (Fig. 2c) with the only exception of *A. baumannii* and *P. vulgaris* against which rifampicin and cefotaxime exhibited higher antibacterial activities with ZOI 33 and 36 mm, respectively. Rifampicin exhibited significantly higher (*P *< 0.001) antibacterial activity compared to other antibiotics against *A. baumannii*. Moxifloxacin and ciprofloxacin mostly exhibited potent antibacterial activities showing slight variation with ofloxacin (positive control) against clinical isolates. Conversely, in reference strains, moxifloxacin exhibited significantly higher (*P *< 0.001) antibacterial activity compared to all other antibiotics against *E. coli* (ATCC 25922) strain.

Against MDR strains, moxifloxacin, among all the test drugs was the most potent antibiotic exhibiting the highest antibacterial activity with ZOI ~ 47 mm against *P. monteilii* (Fig. 2d) and was significantly higher (*P* < 0.001) compared to all other antibiotics except ciprofloxacin. Overall, ofloxacin (positive control) was the most potent one and exhibited slightly higher antibacterial activity than moxifloxacin and ciprofloxacin with ZOI ~ 47.33 mm against *P. chlororaphis*. Cefotaxime was found to be the least potent among other antibiotics against MDRs (Fig. 2d).

### Minimum inhibitory concentration

Minimum inhibitory concentration (MIC) is the lowest concentration of a drug that inhibits the growth of a specific microorganism. MIC of *J. curcas* seed oil and de-oiled seed cake extracts and each of the selected antibiotics against the selected clinical, MDR and reference strains are given in Tables 1, 2, 3, 4, 5, 6, 7 and 8.

**Table 1:** Minimum inhibitory concentration (MIC) and fractional inhibitory concentration (FIC) of methanolic extract and each antibiotic and their combination against clinical pathogenic and reference bacterial isolates

Strain	Compound	MIC^a^ (mg/mL)/MIC^b^ (µg/mL)		FIC	FICI	Output
		Alone	Combined			
*A. baumannii*	Me/Ctx	100/0.19	0.39/0.195	0.0039/1	1.0	Indifference
	Me/R	100/12.5	12.5/0.39	0.125/0.031	0.15	Synergistic
	Me/Of	100/12.5	12.5/6.25	0.125/0.5	0.6	Indifference
	Me/Cip	100/6.25	12.5/6.25	0.125/1	1.12	Indifference
	Me/Mox	100/6.25	6.25/3.125	0.0625/0.5	0.5	Synergistic
*E. coli*	Me/Ctx	100/100	100/50	1/1	2.0	Indifference
	Me/R	100/100	1.56/0.78	0.0625/0.0156	0.0781.0	Synergistic
	Me/Of	100/50	0.39/3.125	0.0039/0.0625	0.06	Synergistic
	Me/Cip	100/100	6.25/0.195	0.0625/0.0039	0.0664	Synergistic
	Me/Mox	100/3.125	25/12.5	0.25/8.01	4.25	Antagonistic
*E. faecalis*	Me/Ctx	100/50	100/50	1/1	2.0	Indifference
	Me/R	100/100	6.25/3.125	0.0625/0.031	0.09	Synergistic
	Me/Of	100/12.5	25/0.39	0.25/0.0312	0.28	Synergistic
	Me/Cip	100/50	0.78/12.5	0.0078/0.25	0.25	Synergistic
	Me/Mox	100/1.56	25/12.5	0.25/8.01	8.26	Antagonistic
*P. vulgaris*	Me/Ctx	100/0.19	12.5/6.25	0.125/32.05	32.17	Antagonistic
	Me/R	100/6.25	12.5/6.25	0.125/1	1.125	Indifference
	Me/Of	100/12.5	12.5/3.125	0.125/0.25	0.375	Synergistic
	Me/Cip	100/6.25	6.25/6.25	0.0625/1	1.062	Indifference
	Me/Mox	100/6.25	100/50	1/8	8.26	Antagonistic
*S. aureus*	Me/Ctx	100/50	0.78/0.39	0.0078/0.0078	0.0156	Synergistic
	Me/R	100/6.25	0.39/0.195	0.0039/0.0312	0.0351	Synergistic
	Me/Of	100/50	0.39/1.56	0.0039/64.10	64.106	antagonistic
	Me/Cip	100/12.5	3.125/0.39	0.0312/0.0312	0.062	Synergistic
	Me/Mox	100/3.125	3.125/1.56	0.0312/0.49	0.53	Synergistic
*P. aeruginosa*	Me/Ctx	100/50	50/3.125	0.06/0.06	0.1	Synergistic
	Me/R	100/6.25	100/1.56	0.03/0.2	0.2	Synergistic
	Me/Of	100/12.5	25/50	0.003/4	4.0039	Antagonism
	Me/Cip	100/3.125	100/0.39	1/0.1	1.1	Indifference
	Me/Mox	100/0.39	25/3.125	0.06/8	8	Antagonistic
*S. aureus* (ATCC)	Me/Ctx	50/50	0.78/0.39	0.008/256	0.023	Synergistic
	Me/R	50/6.25	0.39/0.195	0.007/0.03	0.03	Synergistic
	Me/Of	50/3.125	0.39/0.195	0.007/0.06	0.07	Synergistic
	Me/Cip	50/1.56	1.56/0.78	0.03/0.5	0.5	Synergistic
	Me/Mox	50/0.78	12.5/6.25	0.12/8	8.12	Antagonistic
*E. coli* (ATCC)	Me/Ctx	100/0.78	0.78/0.39	0.007/0.5	0.5	Synergistic
	Me/R	100/6.25	1.56/0.78	0.01/0.12	0.1	Synergistic
	Me/Of	100/3.125	0.097/0.048	0.0009/0.01	0.01	Synergistic
	Me/Cip	100/3.125	0.78/0.39	0.007/0.12	0.1	Synergistic
	Me/Mox	100/0.39	12.5/6.25	0.12/16	16	Antagonistic

*MIC*^a^ minimal inhibitory concentration for methanolic extract when applied alone, *MIC*^b^ minimal inhibitory concentration for antibiotic when applied alone, *Me* methanolic extract, *Ctx* cefotaxime, *R* rifampicin, *Of* ofloxacin, *Cip* ciprofloxacin, *Mox* moxifloxacin, *ND* not determined

**Table 2:** Minimum inhibitory concentration (MIC) and fractional inhibitory concentration (FIC) of *n*-hexane extract, each antibiotic and their combination against clinical pathogenic and reference bacterial isolates

Strain	Compound	MIC^a^ (mg/mL)/MIC^b^ (µg/mL)		FIC	FICI	Output
		Alone	Combined			
*A. baumannii*	*n*-hex/Ctx	100/0.19	1.56/0.78	0.01/4.11	4.1	Antagonistic
	*n*-hex/R	100/6.25	12.5/6.25	0.1/1	1.12	Indifference
	*n*-hex/Of	100/12.5	1.56/0.78	0.01/0.06	0.07	Synergistic
	*n*-hex/Cip	100/6.25	1.56/0.78	0.01/0.1	0.1	Synergistic
	*n*-hex/Mox	100/6.25	12.5/6.25	0.1/1	1.12	Indifference
*E. coli*	*n*-hex/Ctx	100/100	3.125/1.56	0.03/0.01	0.04	Synergistic
	*n*-hex/R	100/100	0.78/0.39	0.007/0.003	0.01	Synergistic
	*n*-hex/Of	100/50	100/50	1/1	2.0	Indifference
	*n*-hex/Cip	100/100	1.56/0.78	0.01/0.007	0.02	Synergistic
	*n*-hex/Mox	100/3.125	25/12.5	0.25/4	4.25	Antagonistic
*E. faecalis*	*n*-hex/Ctx	–/50	100/50	–/1	ND	ND
	*n*-hex/R	–/100	100/50	–/0.5	ND	ND
	*n*-hex/Of	–/12.5	100/50	–/4	ND	ND
	*n*-hex/Cip	–/50	6.25/3.125	–/0.002	ND	ND
	*n*-hex/Mox	–/1.56	25/12.5	–/8.01	ND	ND
*P. vulgaris*	*n*-hex/Ctx	100/0.19	1.56/0.78	0.01/0.1	0.1	Synergistic
	*n*-hex/R	100/6.25	0.78/0.39	0.007/0.06	0.07	Synergistic
	*n*-hex/Of	100/12.5	0.78/0.39	0.007/0.03	0.03	Synergistic
	*n*-hex/Cip	100/6.25	0.78/0.39	0.007/0.06	0.07	Synergistic
	*n*-hex/Mox	100/6.25	100/50	1/8	9.0	Antagonistic
*S. aureus*	*n*-hex/Ctx	–/50	–/–	–/–	ND	ND
	*n*-hex/R	–/6.25	100/50	–/8	ND	ND
	*n*-hex/Of	–/50	100/50	–/1	ND	ND
	*n*-hex/Cip	–/12.5	–/–	–/–	ND	ND
	*n*-hex/Mox	–/3.125	12.5/6.25	–/2	ND	ND
*P. aeruginosa*	*n*-hex/Ctx	200/50	12.5/6.25	0.06/0.1	0.1	Synergistic
	*n*-hex/R	200/6.25	3.125/1.56	0.01/0.2	0.2	Synergistic
	*n*-hex/Of	200/12.5	6.25/3.125	0.03/0.25	0.2	Synergistic
	*n*-hex/Cip	200/3.125	3.125/1.56	0.03/0.4	0.5	Synergistic
	*n*-hex/Mox	200/0.39	6.25/3.125	0.03/8.01	8.04	Antagonistic
*S. aureus (ATCC)*	*n*-hex/Ctx	100/100	25/12.5	0.25/8	8.25	Antagonistic
	*n*-hex/R	100/6.25	6.25/3.125	0.06/0.5	0.5	Synergistic
	*n*-hex/Of	100/3.125	100/50	1/16	17	Antagonistic
	*n*-hex/Cip	100/1.56	0.097/0.048	0.0009/0.03	0.03	Synergistic
	*n*-hex/Mox	100/0.78	6.25/3.125	0.06/4	4.06	Antagonistic
*E. coli (ATCC)*	*n*-hex/Ctx	100/0.78	3.125/1.56	0.03/0.5	0.5	Synergistic
	*n*-hex/R	100/6.25	0.39/0.195	0.003/0.3	0.03	Synergistic
	*n*-hex/Of	100/3.12	0.39/0.195	0.003/0.06	0.06	Synergistic
	*n*-hex/Cip	100/3.12	0.78/0.39	0.007/0.1	0.1	Synergistic
	*n*-hex/Mox	100/0.39	6.25/3.125	0.06/8	8.0	Antagonistic

*MIC*^a^ minimal inhibitory concentration for *n*-hexane extract when applied alone, *MIC*^b^ minimal inhibitory concentration for antibiotic when applied alone, *Me* methanolic extract, *Ctx* cefotaxime, *R* rifampicin, *Of* ofloxacin, *Cip* ciprofloxacin, *Mox* moxifloxacin, *ND* not determined

**Table 3:** Minimum inhibitory concentration (MIC) and fractional inhibitory concentration (FIC) of aqueous extract, each antibiotic and their combination against clinical pathogenic and reference bacterial isolates

Strain	Compound	MIC^a^ (mg/mL)/MIC^b^ (µg/mL)		FIC	FICI	Output
		Alone	Combined			
*A. baumannii*	Aq/Ctx	200/0.19	0.195/0.097	0.0009/0.5	0.5	Synergistic
	Aq/R	200/6.25	1.56/0.78	0.007/0.12	0.1	Synergistic
	Aq/Of	200/12.5	3.125/1.56	0.03/0.12	0.1	Synergistic
	Aq/Cip	200/6.25	1.56/0.78	0.007/0.12	0.1	Synergistic
	Aq/Mox	200/6.25	12.5/6.25	0.12/1	1.125	Indifference
*E. coli*	Aq/Ctx	–/100	100/50	–/0.5	ND	ND
	Aq/R	–/100	3.125/1.56	–/0.01	ND	ND
	Aq/Of	–/50	6.25/3.125	–0.06	ND	ND
	Aq/Cip	–/100	6.25/3.125	–/0.03	ND	ND
	Aq/Mox	–/3.125	25/12.5	–/4	ND	ND
*E. faecalis*	Aq/Ctx	–/50	100/50	–/1	ND	ND
	Aq/R	–/100	1.56/0.78	–/0.007	ND	ND
	Aq/Of	–/12.5	1.56/0.78	–/0.06	ND	ND
	Aq/Cip	–/50	3.125/1.56	–/0.03	ND	ND
	Aq/Mox	–/1.56	50/25	–/16	ND	ND
*P. vulgaris*	Aq/Ctx	–/0.19	6.25/3.125	–/0.5	ND	ND
	Aq/R	–/6.25	6.25/3.125	–/0.5	ND	ND
	Aq/Of	–/12.5	0.78/0.39	–/0.03	ND	ND
	Aq/Cip	–/6.25	0.78/0.39	–/0.06	ND	ND
	Aq/Mox	–/6.25	–/–	–/–	ND	ND
*S. aureus*	Aq/Ctx	–/50	100/50	–/1	ND	ND
	Aq/R	–/6.25	100/50	–/8	ND	ND
	Aq/Of	–/100	6.25/3.125	–/0.25	ND	ND
	Aq/Cip	–/12.5	50/25	–/2	ND	ND
	Aq/Mox	–/3.125	50/25	–/8	ND	ND
*P. aeruginosa*	Aq/Ctx	200/50	100/50	0.5/1	1.5	Indifference
	Aq/R	200/6.25	12.5/6.25	0.06/1	1.06	Indifference
	Aq/Of	200/12.5	12.5/6.25	0.06/0.5	0.5	Synergistic
	Aq/Cip	200/3.125	25/12.5	0.12/4	4.1	Antagonistic
	Aq/Mox	200/0.39	6.25/3.125	0.03/8	8.0	Antagonistic
*S. aureus (ATCC)*	Aq/Ctx	200/100	100/50	0.5/0.5	1.0	Indifference
	Aq/R	200/6.25	100/50	0.5/8	8.5	Antagonistic
	Aq/Of	200/3.12	3.125/1.56	0.5/0.4	0.9	Additive
	Aq/Cip	200/1.56	12.5/6.25	0.06/4	4.06	Antagonistic
	Aq/Mox	200/0.78	12.5/6.25	0.06/8	8.0	Antagonistic
*E. coli (ATCC)*	Aq/Ctx	–/0.78	1.56/0.78	–/1	ND	ND
	Aq/R	–/6.25	1.56/0.78	–/0.1	ND	ND
	Aq/Of	–/3.12	3.125/1.56	–/0.4	ND	ND
	Aq/Cip	–/3.12	1.56/0.78	–/0.2	ND	ND
	Aq/Mox	–/0.39	12.5/6.25	–/16	ND	ND

*MIC*^a^ minimal inhibitory concentration for aqueous extract when applied alone, *MIC*^b^ minimal inhibitory concentration for antibiotic when applied alone, *Me* methanolic extract, *Ctx* cefotaxime, *R* rifampicin, *Of* ofloxacin, *Cip* ciprofloxacin, *Mox* moxifloxacin, *ND* not determined

**Table 4:** Minimum inhibitory concentration (MIC) and fractional inhibitory concentration (FIC) of seed oil each antibiotic and their combination against clinical pathogenic and reference bacterial isolates

Strain	Compound	MIC^a^ (mg/mL)/MIC^b^ (µg/mL)		FIC	FICI	Output
		Alone	Combined			
*A. baumannii*	Oil/Ctx	100/0.19	0.39/0.195	0.0039/1.026	1.03	Synergistic
	Oil/R	100/6.25	0.78/0.39	0.0078/0.062	0.07	Antagonistic
	Oil/Of	100/12.5	6.25/3.125	0.25/0.25	0.5	Synergistic
	Oil/Cip	100/6.25	6.25/3.125	0.031/0.5	0.5	Synergistic
	Oil/Mox	100/6.25	50/25	0.5/2	4.5	Antagonistic
*E. coli*	Oil/Ctx	200/100	6.25/3.125	0.031/0.031	0.06	Synergistic
	Oil/R	200/100	0.78/0.39	0.0039/0.0039	0.0078	Synergistic
	Oil/Of	200/50	0.78/12.5	0.125/0.25	0.3	Synergistic
	Oil/Cip	200/100	25/0.195	0.0078/0.0039	0.01	Synergistic
	Oil/Mox	200/3.125	12.5/6.25	0.125/2	2.12	Indifference
*E. faecalis*	Oil/Ctx	100/50	1.56/0.78	0.015/0.015	0.03	Synergistic
	Oil/R	100/100	1.56/0.78	0.015/0.0078	0.02	Synergistic
	Oil/Of	100/12.5	1.56/1.56	0.015/0.124	0.1	Synergistic
	Oil/Cip	100/50	1.56/0.78	0.015/0.015	0.03	Synergistic
	Oil/Mox	100/1.56	1.56/3.125	0.06/2	2.0	Indifference
*P. vulgaris*	Oil/Ctx	100/0.19	0.39/0.195	0.0039/0.03	0.03	Synergistic
	Oil/R	100/6.25	3.125/1.56	0.03/0.24	0.2	Synergistic
	Oil/Of	100/12.5	3.125/0.195	0.03/0.015	0.04	Synergistic
	Oil/Cip	100/6.25	0.78/1.56	–/0.24	ND	ND
	Oil/Mox	100/6.25	100/50	–/16	ND	ND
*S. aureus*	Oil/Ctx	–/50	3.125/1.56	–/0.03	ND	ND
	Oil/R	–/6.25	1.56/0.78	–/0.12	ND	ND
	Oil/Of	–/100	1.56/0.78	–/0.06	ND	ND
	Oil/Cip	–/12.5	0.195/0.097	0.0009/0.0077	0.008	Synergistic
	Oil/Mox	–/3.125	12.5/6.25	0.03/2	2.0	Indifference
*P. aeruginosa*	Oil/Ctx	200/50	0.39/0.195	0.001/0.0039	0.005	Synergistic
	Oil/R	200/6.25	12.5/6.25	0.06/1	1.0	Indifference
	Oil/Of	200/12.5	6.25/3.125	0.03/0.1	0.1	Synergistic
	Oil/Cip	200/3.125	3.125/3.125	2/1	3.0	Indifference
	Oil/Mox	200/0.39	3.125/1.56	0.25/4	4.2	Antagonistic
*S. aureus(ATCC)*	Oil/Ctx	–/100	0.78/0.39	–/0.003	ND	ND
	Oil/R	–/6.25	0.195/0.097	–/0.01	ND	ND
	Oil/Of	–/3.125	0.78/0.39	–/0.12	ND	ND
	Oil/Cip	–/1.56	0.195/0.0485	–/0.03	ND	ND
	Oil/Mox	–/0.781	3.125/1.56	–/1.99	ND	ND
*E. coli (ATCC)*	Oil/Ctx	100/0.781	0.78/0.39	0.0078/0.5	0.5	Synergistic
	Oil/R	100/6.25	0.78/0.39	0.0078/0.06	0.07	Synergistic
	Oil/Of	100/3.125	0.095/0.0485	0.00097/0.015	0.01	Synergistic
	Oil/Cip	100/3.125	0.095/0.0485	0.0097/0.01	0.01	Synergistic
	Oil/Mox	100/0.39	6.25/3.125	0.06/8.01	8.0	Antagonistic

*MIC*^a^ minimal inhibitory concentration for seed oil when applied alone, *MIC*^b^ minimal inhibitory concentration for antibiotic when applied alone, *Me* methanolic extract, *Ctx* cefotaxime, *R* rifampicin, *Of* ofloxacin, *Cip* ciprofloxacin, *Mox* moxifloxacin, *ND* not determined

**Table 5:** Minimum inhibitory concentration (MIC) and fractional inhibitory concentration (FIC) of methanolic extract, each antibiotic alone and their combination against MDR pathogenic bacterial isolates

Strain	Compound	MIC^a^ (mg/mL)/MIC^b^ (µg/mL)		FIC	FICI	Output
		Alone	Combined			
MRSA1	Me/Ctx	50/100	50/25	0.5/2	2.5	Indifference
	Me/R	50/100	100/50	0.5/2	2.5	Indifference
	Me/Of	50/100	100/50	0.5/2	2.5	Indifference
	Me/Cip	50/50	25/12.5	0.5/0.25	0.75	Additive
	Me/Mox	50/1.56	25/12.5	0.5/8.01	8.51	Antagonistic
MRSA2	Me/Ctx	50/100	50/25	1/4	5.0	Antagonistic
	Me/R	50/3.125	1.56/0.78	0.03/0.24	0.28	Synergistic
	Me/Of	50/25	100/50	2/2	4.0	Indifference
	Me/Cip	50/50	50/25	1/0.5	1.5	Indifference
	Me/Mox	50/0.781	12.5/6.25	0.25/8	8.25	Antagonistic
MRSA3	Me/Ctx	50/50	50/25	1/0.5	1.5	Indifference
	Me/R	50/3.125	0.19/0.09	0.003/0.006	0.01	Synergistic
	Me/Of	50/50	50/25	1/0.5	1.5	Indifference
	Me/Cip	50/12.5	25/12.5	0.5/1	1.5	Indifference
	Me/Mox	50/0.39	3.125/1.56	0.06/4	4.06	Antagonistic
MRSA4	Me/Ctx	100/50	1.56/0.78	0.01/0.01	0.03	Synergistic
	Me/R	100/3.125	0.78/0.39	0.007/0.12	0.13	Synergistic
	Me/Of	100/1.56	0.78/0.78	0.007/0.5	0.5	Synergistic
	Me/Cip	100/3.125	1.56/0.39	0.01/0.12	0.14	Synergistic
	Me/Mox	100/0.781	25/12.5	0.25/16	16.25	Antagonistic
MRSA5	Me/Ctx	100/3.125	100/50	1/16	17	Antagonistic
	Me/R	100/3.125	0.78/0.39	0.007/0.12	0.13	Synergistic
	Me/Of	100/100	50/25	0.5/4	4.5	Antagonistic
	Me/Cip	100/50	50/25	0.5/0.5	1.0	Indifference
	Me/Mox	100/1.56	6.25/3.125	0.06/2	2.06	Indifference
*A. baumannii* MDR	Me/Ctx	100/6.25	3.12/1.56	0.03/0.24	0.2	Synergistic
	Me/R	100/3.125	0.39/0.195	0.003/0.06	0.06	Synergistic
	Me/Of	100/0.78	1.56/0.78	0.01/1	1.01	Indifference
	Me/Cip	100/6.25	0.39/0.195	0.003/0.03	0.03	Synergistic
	Me/Mox	100/1.56	6.25/3.125	0.06/2	2.0	Indifference
*K. pneumoniae*	Me/Ctx	100/100	100/50	1/2	3.0	Indifference
	Me/R	100/100	50/25	0.5/0.25	0.75	Additive
	Me/Of	100/25	50/25	0.5/0.03	0.5	Synergistic
	Me/Cip	100/50	50/25	0.5/0.003	0.5	Synergistic
	Me/Mox	100/1.56	12.5/6.25	0.12/4	4.13	Antagonistic
*P. chlororaphis*	Me/Ctx	100/–	25/12.5	0.25/–	ND	ND
	Me/R	100/100	25/12.5	0.25/0.125	0.38	Synergistic
	Me/Of	100/6.25	100/50	1/0.12	1.12	Indifference
	Me/Cip	100/3.125	12.5/6.25	0.12/0.06	0.18	Synergistic
	Me/Mox	100/1.56	3.125/1.56	0.03/1	1.03	Indifference
*P. monteilii*	Me/Ctx	–/100	0.39/0.195	–/512	ND	ND
	Me/R	–/–	0.39/0.195	–/–	ND	ND
	Me/Of	–/100	0.78/0.39	–/256	ND	ND
	Me/Cip	–/50	0.39/0.195	–/0.003	ND	ND
	Me/Mox	–/0.78	100/50	–/64	ND	ND

*MIC*^a^ minimal inhibitory concentration for de-oiled seed extract when applied alone, *MIC*^b^ minimal inhibitory concentration for antibiotic when applied alone, *Me* methanolic extract, *Ctx* cefotaxime, *R* rifampicin, *Of* ofloxacin, *Cip* ciprofloxacin, *Mox* moxifloxacin, *ND* not determined

**Table 6:** Minimum inhibitory concentration (MIC) and fractional inhibitory concentration (FIC) of *n*-hexane extract, each antibiotic alone and their combination against MDR pathogenic bacterial isolates

Strain	Compound	MIC^a^ (mg/mL)/MIC^b^ (µg/mL)		FIC	FICI	Output
		Alone	Combined			
MRSA1	*n*-hex/Ctx	100/100	12.5/6.25	0.1/0.06	0.1	Synergistic
	*n*-hex/R	100/100	100/50	1/0.5	1.5	Indifference
	*n*-hex/Of	100/100	100/50	1/0.5	1.5	Indifference
	*n*-hex/Cip	100/50	100/50	2/1	3	Indifference
	*n*-hex/Mox	100/1.56	100/50	1/32	33.05	Antagonistic
MRSA2	*n*-hex/Ctx	–/100	6.25/3.125	–/0.03	ND	ND
	*n*-hex/R	–/3.125	12.5/6.25	–/2	ND	ND
	*n*-hex/Of	–/25	1.56/0.78	–/0.03	ND	ND
	*n*-hex/Cip	–/50	0.39/0.195	0.007/0.003	0.01	Synergistic
	*n*-hex/Mox	–/0.78	25/12.5	–/16	ND	ND
MRSA3	*n*-hex/Ctx	100/50	25/12.5	0.25/0.25	0.5	Synergistic
	*n*-hex/R	100/3.125	1.56/0.78	0.01/0.2	0.2	Synergistic
	*n*-hex/Of	100/50	100/50	0.01/1	1.01	Indifference
	*n*-hex/Cip	100/12.5	100/50	2/4	6.0	Antagonistic
	*n*-hex/Mox	100/0.39	6.25/3.125	0.1/8	8.13	Antagonistic
MRSA4	*n*-hex/Ctx	100/50	12.5/6.25	0.1/0.1	0.25	Synergistic
	*n*-hex/R	100/3.125	3.125/1.56	0.03/0.4	0.5	Synergistic
	*n*-hex/Of	100/1.56	1.56/0.78	1/0.5	1.5	Indifference
	*n*-hex/Cip	100/3.125	1.56/0.78	0.01/0.2	0.2	Synergistic
	*n*-hex/Mox	100/0.78	100/50	1/64	65	Antagonistic
MRSA5	*n*-hex/Ctx	100/3.125	6.25/3.125	0.06/1	1.06	Indifference
	*n*-hex/R	100/3.125	0.39/0.195	0.003/0.06	0.06	Synergistic
	*n*-hex/Of	100/100	100/50	0.01/0.5	0.5	Synergistic
	*n*-hex/Cip	100/50	100/50	1/1	2.0	Indifference
	*n*-hex/Mox	100/1.56	6.253.125	0.6/2	2.0	Indifference
*baumannii* MDR	*n*-hex/Ctx	–/6.25	3.125/1.56	–/0.2	ND	ND
	*n*-hex/R	–/3.125	0.39/0.195	–/0.06	ND	ND
	*n*-hex/Of	–/0.78	1.56/0.78	–/1	ND	ND
	*n*-hex/Cip	–/6.25	1.56/0.78	–/0.1	ND	ND
	*n*-hex/Mox	–/1.56	6.25/3.125	–/2	ND	ND
*K. pneumoniae*	*n*-hex/Ctx	–/100	100/50	–/0.5	ND	ND
	*n*-hex/R	–/100	100/50	–/0.5	ND	ND
	*n*-hex/Of	–/25	100/50	–/2	ND	ND
	*n*-hex/Cip	–/50	100/50	–/1	ND	ND
	*n*-hex/Mox	–/1.56	12.5/6.25	–/4	ND	ND
*P. chlororaphis*	*n*-hex/Ctx	–/–	–/50	–/–	ND	ND
	*n*-hex/R	–/100	100/50	–/0.5	ND	ND
	*n*-hex/Of	–/6.25	100/50	–/8	ND	ND
	*n*-hex/Cip	–/3.125	50/25	–/8	ND	ND
	*n*-hex/Mox	–/1.56	12.5/6.25	–/4	ND	ND
*P. monteilii*	*n*-hex/Ctx	–/100	0.78/0.39	–/–	ND	ND
	*n*-hex/R	–/–	0.78/0.39	–/–	ND	ND
	*n*-hex/Of	–/100	1.56/0.78	–/0.007	ND	ND
	*n*-hex/Cip	–/50	1.56/0.78	–/0.01	ND	ND
	*n*-hex/Mox	–/0.78	100/50	–/64	ND	ND

*MIC*^a^ minimal inhibitory concentration for *n*-hexane extract when applied alone, *MIC*^b^ minimal inhibitory concentration for antibiotic when applied alone, *Me* methanolic extract, *Ctx* cefotaxime, *R* rifampicin, *Of* ofloxacin, *Cip* ciprofloxacin, *Mox* moxifloxacin, *ND* not determined

**Table 7:** Minimum inhibitory concentration (MIC) and fractional inhibitory concentration (FIC) of aqueous extract, each antibiotic alone and their combination against MDR pathogenic bacterial isolates

Strain	Compound	MIC^a^ (mg/mL)/MIC^b^ (µg/mL)		FIC	FICI	Output
		Alone	Combined			
MRSA1	Aq/Ctx	–/100	50/25	–/0.25	ND	ND
	Aq/R	–/100	1.56/0.78	–/0.007	ND	ND
	Aq/Of	–/100	100/50	–/0.5	ND	ND
	Aq/Cip	–/50	100/50	–/1	ND	ND
	Aq/Mox	–/1.56	100/50	–/32	ND	ND
MRSA2	Aq/Ctx	–/100	100/50	–/0.5	ND	ND
	Aq/R	–/3.125	0.78/0.39	–/0.1	ND	ND
	Aq/Of	–/25	100/50	–/2	ND	ND
	Aq/Cip	–/50	100/50	–/1	ND	ND
	Aq/Mox	–/0.78	25/12.5	–/16	ND	ND
MRSA3	Aq/Ctx	200/50	100/50	0.5/1	1.5	Indifference
	Aq/R	200/3.13	0.39/0.195	0.001/0.06	0.06	Synergistic
	Aq/Of	200/50	100/50	0.5/1	1.5	Indifference
	Aq/Cip	200/12.5	100/50	2/4	6	Antagonistic
	Aq/Mox	200/0.39	12.5/6.25	0.25/16	16.2	Antagonistic
MRSA4	Aq/Ctx	–/50	100/50	–/1	ND	ND
	Aq/R	–/3.125	100/50	–/16	ND	ND
	Aq/Of	–/1.56	100/50	–/32	ND	ND
	Aq/Cip	–/3.125	100/50	–/16	ND	ND
	Aq/Mox	–/0.78	50/25	–/32	ND	ND
MRSA5	Aq/Ctx	200/3.13	100/50	0.5/16	16.5	Antagonistic
	Aq/R	200/3.13	6.25/3.125	0.03/1	1.03	Indifference
	Aq/Of	200/100	12.5/6.25	0.03/0.06	0.09	Synergistic
	Aq/Cip	200/50	12.5/6.25	0.06/0.1	0.18	Synergistic
	Aq/Mox	200/1.56	6.25/3.125	0.06/2	2.0	Indifference
*A. baumannii* MDR	Aq/Ctx	–/6.25	100/50	–/8	ND	ND
	Aq/R	–/3.125	0.78/0.39	–/0.1	ND	ND
	Aq/Of	–/0.78	100/50	–/64	ND	ND
	Aq/Cip	–/6.25	100/50	–/8	ND	ND
	Aq/Mox	–/1.56	6.25/3.125	–/2	ND	ND
*Klebsiella pneumoniae*	Aq/Ctx	–/100	100/50	–/0.5	ND	ND
	Aq/R	–/100	100/50	–/0.5	ND	ND
	Aq/Of	–/25	100/50	–/2	ND	ND
	Aq/Cip	–/50	100/50	–/1	ND	ND
	Aq/Mox	–/1.56	12.5/6.25	–/4	ND	ND
*P. chlororaphis*	Aq/Ctx	–/–	–/–	–/–	ND	ND
	Aq/R	–/100	100/50	–/0.5	ND	ND
	Aq/Of	–/6.25	50/25	–/4	ND	ND
	Aq/Cip	–/3.125	50/25	–/8	ND	ND
	Aq/Mox	–/1.56	6.25/3.125	–/2	ND	ND
*P. monteilii*	Aq/Ctx	–/100	100/50	–/0.5	ND	ND
	Aq/R	–/–	1.56/0.78	–/–	ND	ND
	Aq/Of	–/100	1.56/0.78	–/0.007	ND	ND
	Aq/Cip	–/50	0.78/0.39	–/0.007	ND	ND
	Aq/Mox	–/0.78	100/50	–/64	ND	ND

*MIC*^a^ minimal inhibitory concentration for aqueous extract when applied alone, *MIC*^b^ minimal inhibitory concentration for antibiotic when applied alone, *Me* methanolic extract, *Ctx* cefotaxime, *R* rifampicin, *Of* ofloxacin, *Cip* ciprofloxacin, *Mox* moxifloxacin, *ND* not determined

**Table 8:** Minimum inhibitory concentration (MIC) and fractional inhibitory concentration (FIC) of seed oil, each antibiotic alone and their combination against MDR pathogenic bacterial isolates

Strain		MIC^a^ (mg/mL)/MIC^b^ (µg/mL)		FIC	FICI	Output
	Compound	Alone	Combined			
MRSA1	Oil/Ctx	50/100	100/50	2/0.5	2.5	Indifference
	Oil/R	50/100	100/50	–/0.5	ND	ND
	Oil/Of	50/100	3.125/1.56	0.06/0.01	0.07	Synergistic
	Oil/Cip	50/50	100/50	–/1	ND	ND
	Oil/Mox	50/1.56	25/12.5	–/8.01	ND	ND
MRSA2	Oil/Ctx	–/100	25/12.5	–/0.1	ND	ND
	Oil/R	–/3.125	25/12.5	–/4	ND	ND
	Oil/Of	–/25	1.56/0.78	–/0.06	ND	ND
	Oil/Cip	–/50	1.56/0.78	–/0.03	ND	ND
	Oil/Mox	–/0.781	6.25/3.125	–/4	ND	ND
MRSA3	Oil/Ctx	–/50	3.125/1.56	–/0.03	ND	ND
	Oil/R	–/3.125	3.125/1.56	–/0.03	ND	ND
	Oil/Of	–/50	50/25	–/0.0078	ND	ND
	Oil/Cip	–/12.5	25/12.5	–/0.03	ND	ND
	Oil/Mox	–/0.39	3.125/1.56	–/8.01	ND	ND
MRSA4	Oil/Ctx	–/50	0.39/0.195	–/0.003	ND	ND
	Oil/R	–/3.125	3.125/1.56	–/0.49	ND	ND
	Oil/Of	–/1.56	1.56/0.78	–/0.5	ND	ND
	Oil/Cip	–/3.125	3.125/1.56	–/0.49	ND	ND
	Oil/Mox	–/0.781	100/50	–/1.99	ND	ND
MRSA5	Oil/Ctx	–/3.125	50/25	–/8	ND	ND
	Oil/R	–/3.125	0.78/0.39	–/0.12	ND	ND
	Oil/Of	–/100	12.5/6.25	–/0.06	ND	ND
	Oil/Cip	–/50	100/50	–/1	ND	ND
	Oil/Mox	–/1.56	6.25/3.125	–/32	ND	ND
*A. baumannii* MDR	Oil/Ctx	–/6.25	3.125/1.56	–/0.24	ND	ND
	Oil/R	–/3.125	0.195/0.097	–/0.03	ND	ND
	Oil/Of	–/0.78	0.78/0.3	–/0.5	ND	ND
	Oil/Cip	–/6.25	0.78/0.39	–/0.06	ND	ND
	Oil/Mox	–/1.56	3.125/1.56	–/1	ND	ND
*K. pneumoniae*	Oil/Ctx	–/100	100/50	–/0.5	ND	ND
	Oil/R	–/100	100/50	–/0.5	ND	ND
	Oil/Of	–/25	100/50	–/2	ND	ND
	Oil/Cip	–/50	12.5/6.25	–/0.12	ND	ND
	Oil/Mox	–/1.56	6.25/3.125	–/2	ND	ND
*P. chlororaphis*	Oil/Ctx	–/–	–/–	–/–	ND	ND
	Oil/R	–/100	1.56/0.78	–/0.0078	ND	ND
	Oil/Of	–/6.25	100/50	–/8	ND	ND
	Oil/Cip	–/3.125	3.125/1.56	–/0.4	ND	ND
	Oil/Mox	–/1.56	6.253.125	–/2	ND	ND
*P. monteilii*	Oil/Ctx	–/100	6.25/3.125	–/0.03	ND	ND
	Oil/R	–/–	0.78/0.39	–/–	ND	ND
	Oil/Of	–/100	0.78/0.39	–/0.0039	ND	ND
	Oil/Cip	–/50	0.78/0.39	–/0.0078	ND	ND
	Oil/Mox	–/0.781	100/50	–/64	ND	ND

*MIC*^a^ minimal inhibitory concentration for seed oil when applied alone, *MIC*^b^ minimal inhibitory concentration for antibiotic when applied alone, *Me* methanolic extract, *Ctx* cefotaxime, *R* rifampicin, *Of* ofloxacin, *Cip* ciprofloxacin, *Mox* moxifloxacin, *ND* not determined

When screened individually, methanolic extract exhibited higher antibacterial potential at lower concentration compared to seed oil, *n*-hexane and aqueous extracts against various clinical, MDR and reference bacterial strains. Individually, methanolic extract was found to be the most potent with the least MIC ~ 50 mg/mL against methicillin resistant *S. aureus* (MRSA1, MRSA2 and MRSA3) and *S. aureus* ATCC25923 strains (Table 5). A similar activity (least MIC ~ 50 mg/mL) was observed for seed oil only against MRSA1 strain (Table 8). On the other hand, seed oil and aqueous extract were determined mostly inactive at any concentration against clinical, MDR bacterial strains shown in (Tables 3, 4, 7 and 8). Even the aqueous extract did not exhibit any activity against *E. coli* ATCC25922 reference strain. The MICs for *J. curcas* seed oil, methanolic, *n*-hexane and aqueous extracts were found in 50 to 200 mg/mL range against clinical, MDR and reference bacterial strains (Tables 1, 2, 3, 4, 5, 6, 7, 8). Among all the antibiotics, when evaluated for their antibacterial activity individually, cefotaxime was found to be the most potent with an MIC value of 0.195 µg/mL against clinical isolates, including *A. baumannii* and *P. vulgaris* (Tables 1, 2, 3, 4). On the other hand, rifampicin was found to be the least potent drug, exhibiting no antibacterial activity with an MIC of less than 3.125 µg/mL against any bacterial strain (Tables 1, 2, 3, 4, 5, 6, 7, 8). Overall, for antibiotics when applied individually, MICs were found to be in the range of 0.19 to 100 µg/mL against various bacterial strains (Tables 1, 2, 3, 4, 5, 6, 7, 8).

When screened in combination, the antimicrobial potential of extracts was enhanced compared to individual extract’s MICs. For extracts in combinations, the methanolic and *n*-hexane extracts in combination with ofloxacin and ciprofloxacin were found highly potent with least MIC (0.097 mg/mL) against *E. coli* (ATCC25922) and *S. aureus* (ATCC25923), respectively. Overall, the MIC for extracts in combination with antibiotics ranged from 0.097 to 100 mg/mL against all clinical MDR and reference bacterial strains (Tables 1, 2, 3, 4, 5, 6, 7, 8). Moreover, in case of antibiotics combinations, the most potent activities were exhibited with MIC 0.049 µg/mL against reference strains. Ciprofloxacin in combination with seed oil and *n*-hexane extract exhibited MIC 0.049 µg/mL against *S. aureus* (ATCC25923). Similarly, ofloxacin in combination with seed oil or methanolic extract and ciprofloxacin in combination with seed oil exhibited MIC (0.049 µg/mL) against *E. coli* (ATCC25922) strain. Other antibiotics in combinations such as rifampicin plus methanolic extract and cefotaxime plus aqueous extracts were found to be potent with MIC 0.097 µg/mL against MRSA3 and *A. baumannii*, respectively. Similarly, rifampicin plus seed oil also exhibited antibacterial activity with MIC 0.097 µg/mL against *A. baumannii* MDR and MRSA3 and the same activity was also exhibited by ciprofloxacin in combination with seed oil against *S. aureus*.

### Fractional inhibitory concentration

The combinatorial drug effects were evaluated using fractional inhibitory concentration index (FICI) according to the criteria reported earlier (Hossain et al. 2016), where the effect of combination therapy is considered as “synergistic” if the FICI is ≤ 0.5; “additive” if FICI is > 0.5 and ≤ 1, “indifferent” if FICI is > 1 and ≤ 4 and antagonistic if FICI > 4. Among all the combinations, methanolic extract in combination with rifampicin exhibited the highest synergistic effect (FICI ≤ 0.5) against the isolates including *A. baumannii*, *E. coli, E. faecalis, S. aureus,* and *P. aeruginosa*, methicillin resistant *S. aureus* (MRSA2, MRSA3, MRSA4 and MRSA5), *A. baumannii* (MDR strain), *P. chlororaphis*, *E. coli* ATCC25922 and *S. aureus* ATCC25923 (Tables 1 and 5). The rate of synergism against clinical, MDR and reference bacterial strains remained the highest for methanolic extract in combination with rifampicin (15.29%), followed by ciprofloxacin plus methanolic (11.76%), ofloxacin plus methanolic/seed oil (8.24%), cefotaxime plus *n*-hexane (8.24%) and moxifloxacin plus methanolic extract (2.35%). Among all the isolates, *E. coli* (ATCC25922) was found to be the most susceptible strain in combinatorial therapy. Methanolic, *n*-hexane extracts and seed oil in combination with rifampicin, ciprofloxacin, ofloxacin and cefotaxime showed synergistic effects against *E. coli* (ATCC25922) (Tables 1, 2 and 4). Among the selected antibiotics, moxifloxacin in combination with all extracts was found to have least synergistic while highest antagonistic effects (7.35%) against clinical, MDR and reference strains (Tables 1, 2, 3, 4, 5, 6, 7, 8) but its effect remained synergistic when applied in combination with methanolic extract against *A. baumannii* and *S. aureus* strains (Table 1). Aqueous extract among others, was the least synergistic rate in combination with any antibiotic (9.41%), showing no synergistic effects against *E. coli*, *E. faecalis*, *P. vulgaris and S. aureus* strains (Table 8). On the other hand, seed oil was comparatively better than aqueous extract and showed 25.88% synergism rate. The seed oil showed strong synergistic effects in combination with cefotaxime against *A. baumannii*, *E. coli, P. vulgaris*, *E. faecalis*, *P. aeruginosa*, and *E. coli* ATCC25922. It was the least potent in combinatorial activities against MDR strains determining none of the interactions but exhibited synergistic and indifferent interactions in combination with ofloxacin and cefotaxime only against MRSA1 (Table 8). Overall, the methanolic, *n*-hexane, aqueous extracts and seed oil in combination with antibiotics against all bacterial strains showed 44.71, 32.94, 9.41 and 25.88% synergism, respectively. In general, the synergistic, indifferent, antagonistic and additive effects by all extracts in combination with all antibiotics against various clinical strains were 28.24, 13.82, 11.76 and 1.76%, respectively (Tables 1, 2, 3, 4, 5, 6, 7, 8).

### Molecular docking

The MurF ligase enzyme was selected as a receptor for unveiling the binding conformation of methanolic extract compounds. MurF ligase enzyme has been an attractive drug target against bacterial pathogens because of its high specificity, selectivity and well determined crystal structure. MurF ligase is involved in the final stage of peptidoglycan synthesis and has been validated as an ideal target for therapeutic compounds. *J. curcas* is highly enriched in long chain fatty acids and other phytochemicals which target the bacterial membranes or cell wall. Therefore, in the present study, MurF ligase was selected as target for phytochemical’s intervention. It was revealed that among all the compounds of the extract, compound beta-monolaurin has the highest affinity for the MurF ligase active pocket with binding energy of − 7.3 kcal/mol (see Additional file 1: Table S10). The compound formed multiple hydrogen and hydrophobic interactions with the active site residues of the MurF ligase enzyme that is the key for the formation of stable complex. The 2-(vinyloxy) propane-1,3-diol, in particular, is involved in three strong hydrogen bondings: each with Ser44, Arg45 and Gln69 that constitute the core active pocket of the enzyme (Ahmad et al. 2017). This compound was further found to be in a position that can antagonistically block the access of natural substrate for MurF active site. The binding interactions and conformation of the compound can be seen in Additional file 1: Figure S5. The compound formed multiple hydrogen and hydrophobic interactions with the active site residues of the MurF ligase enzyme, that are key for the formation stable complex. The acetic acid in particular is involved in two strong hydrogen bonds: each with Ser44 and Asp43 that constitute the core active pocket of the enzyme. This compound was also further found to be posed in a position that can antagonistically blocked the access of natural substrate for MurF active site. Similarly 9,12 octadecadienoic acid present in *n*-hexane extract also exhibited stronger binding affinity for MurF ligase active pocket with binding energy of − 6.2 kcal/mol (see Additional file 1: Table S11). The 9,12 octadecadienoic acid also formed hydrogen and hydrophobic interactions with active site residues of the target protein and bonded to Ser44, Ser43, Phe61 and Leu153 or Leu56. The binding interactions and conformation of the 9,12 octadecadienoic acid can be seen in Additional file 1: Figure S6.

## Discussion

*Jatropha curcas* has traditionally been used in medicine and its biological properties extensively investigated. During the last few decades, numerous biologically and medicinally important phytochemicals including flavonoids, tannins, steroids, saponins, glycosides, cardiac glycosides, volatile oils have been reported in *J. curcas* seed, increasing its medicinal importance (Rachana et al. 2012). Some of the bioactive constituents of this plant have been used to cure various diseases such as coated tongue, dysentery, infertility, gonorrhea, hemorrhoids, skin infections and inflammation (Hassan et al. 2004). Moreover, the phytochemicals play vital roles in plant defense mechanism against different microbial infections (Yadav and Agarwala 2011).

The current study aims to develop a novel strategy towards the discovery of new antibiotics by combining *J. curcas* seed oil and de-oiled seed extracts with commercially available antibiotics against various clinical, MRSA and MDR bacterial strains to combat prevailing antibiotic resistance.

The study included FTIR spectroscopic analyses of *J. curcas* seed oil and methanolic, *n*-hexane and aqueous extracts of its de-oiled seed. Various absorption bands in the FTIR spectra indicated the presence of different biological compounds such as proteins, carbohydrates, lignin, aromatic compounds, alkaloids, esters (phorbol esters and fatty acids methyl esters), amides, cellulose, hemicellulose and fatty acids. Alkaloids, phorbol esters, fatty acids and its methyl esters might be the main antimicrobial components as their antimicrobial activity has been reported earlier (Abdelgadir and Van Staden 2013; Chandrasekaran et al. 2008).

The GC–MS analyses determined the presence of a broad range of bioactive compounds in *J. curcas* extracts. In seed oil and *n*-hexane extract, a number of long chain fatty acids were detected. The antibacterial mechanism of long chain fatty acids is still unknown but the OH groups present in these fatty acids target the bacterial cell membrane (Wojtczak and Wie 1999). Due to their amphipathic nature, fatty acids can solubilize various membrane components such as lipid bilayer and proteins that may lead to cell lysis (Greenway and Dyke 1979). They also affect various cellular processes including electron transport chain, oxidative phosphorylation reaction, enzyme inhibition, production of peroxides and altering electron gradient resulting in the leakage of cellular components from cells and manifests various inhibitory and bactericidal effects (Desbois and Smith 2010).

In the present study, beta-monolaurin (ester of glycerol and lauric acid) and 9-hexadecenal and 1-docosanol, found in the methanolic extract of *J. curcas* de-oiled seed, has previously been reported to have antimicrobial potential that may damage extracellular membrane, denature proteins and DNA or inhibit various macromolecular biosynthesis processes (Mamza et al. 2012; Sheela and Uthayakumari 2013; Skřivanová et al. 2006). Another medicinally important compound, I-(+)-ascorbic acid 2,6-dihexadecanoate, identified in the methanolic extract has strong antioxidant activities and has been used in wound healing (Okwu and Ighodaro 2009). In GC–MS analysis of aqueous extract, a number of bioactive compounds such as 1,4-dithiane, dodecanoic acid, methyl ester, methyl tetradecanoate, vitamin D3, palmitic acid, methyl ester, isopropyl linoleate and di-*n*-octyl phthalate were identified. The antimicrobial potential of the aqueous extract can be attributed to the presence of these compounds (Chandrasekaran et al. 2008). Vitamin D3, identified in aqueous extract, which has the capability to mediate innate immunity in humans and can be used as defense against various infections (Farazi et al. 2017).

In the present study, methanolic extracts among others was found comparatively more potent against clinical, MDR and ATCC bacterial strains (Fig. 2). Individually, methanolic extract exhibited the highest activity against *S. aureus*, *S. aureus* ATCC and MRSA4 among the clinical isolates, reference or MDR strains, respectively. Methanolic extracts of a number of medicinal plants had previously been reported with higher antimicrobial potential compared to *n*-hexane and aqueous extracts (Haq et al. 2016; Tripathi et al. 2016), suggesting its higher biological significance. This study also affirmed the antimicrobial potency of methanolic extract by molecular docking studies that unveiled beta-monolaurin as the best conformation in the active pocket of potential antimicrobial MurF target. Similarly, 9,12 octadecadienoic acid present in *n*-hexane extract also showed strong interaction and affinity with MurF ligase active pocket. In contrast to the previously reported data (Nazzaro et al. 2013), methanolic extract exhibited greater activity against Gram negative than Gram positive clinical isolates. However, some studies revealed that Gram positive strains were less susceptible to bioactive compounds than Gram negative ones because the outer membrane of the latter is not fully impermeable. In contrast, in case of MDR strains, the extracts and seed oil were more active against Gram positive than Gram negative bacteria, probably due to the impermeability of outer membrane of the latter. The results are in close coherence with previously reported data (Kaur and Arora 2009).

The selected antibiotics at initial concentration were found active against most of the clinical and MDR bacterial strains in the following order: moxifloxacin > ciprofloxacin > ofloxacin > cefotaxime > rifampicin.

Combinatorial therapy or synergistic interaction is recommended as an effective strategy to help resolve the issue of antibiotic resistance, cellular toxicity and long-term treatments of the available antibiotics. It can also add to find broad-spectrum antibiotics compared to monotherapies (Marr et al. 2004). In the current study, antibiotics were combined with potent bioactive compounds of the *J. curcas,* aiming to increase their antibacterial potential, overcome resistance and reduce the cost and duration of antimicrobial therapy. When evaluated in combination with *J. curcas* extracts or seed oil, the activity of the selected antibiotics increased (MICs range of 0.097 to 100 mg/mL) as compared to the activity of *J. curcas* extracts or seed oil when screened alone (MIC range of 50 to 200 mg/mL). Individually, methanolic extract remained the most active (MIC 50 mg/mL) amongst the de-oiled seed extracts and seed oil against various methicillin resistant *S. aureus* (MRSA1, MRSA2 and MRSA3) strains. The MIC value of the plant extract below 0.1 mg/mL is considered significant, moderate below or equal to 0.625 mg/mL and weak above 0.625 mg/mL (Kuete 2010). Individually, cefotaxime was the most potent (MIC 0.19 µg/mL) against clinical isolates, *A. baumannii* and *P. vulgaris*. Methanolic or *n*-hexane extracts in combination with ofloxacin or ciprofloxacin against *E. coli* (ATCC25922) or *S. aureus* (ATCC25923) exhibited the highest antibacterial activity (MIC 0.097 mg/mL), respectively. Moreover, in combination with seed oil and *n*-hexane extract, ciprofloxacin exhibited highest activity against *S. aureus* ATCC25923 (MIC 0.045 µg/mL). Ofloxacin in combination with seed oil or methanolic extract and ciprofloxacin with seed oil exhibited a similar activity (MIC 0.049 µg/mL) against *E. coli* ATCC25922 strain, while rifampicin with all extracts exhibited high activity (MIC 0.097 µg/mL) against *A. baumannii* MDR and MRSA3 strains.

The antibiotic/extracts combinations screened as antibacterial agents in this study, were also studied to evaluate their synergistic, indifferent, additive or antagonistic effect that occurs when the antibacterial activity of the drug combination exceeds the sum of the individual drug activities, the activity of both drugs (in combination or individually) remains equal, there is no obvious change in the activity of both drugs (in combination or individually) or the activity of one drug is reduced in the presence of other, respectively (Borisy et al. 2003; Branen and Davidson 2004)]. In combinations, methanolic extract and rifampicin exhibited synergistic rates of 15.29% against selected clinical pathogenic strains. These treatments exhibited the highest synergistic activities against *A. baumannii*, *E. coli, E. faecalis, S. aureus,* and *P. aeruginosa*, methicillin resistant *S. aureus* (MRSA2, MRSA3, MRSA4 and MRSA5), *A. baumannii* (MDR strain), *P. chlororaphis*, *E. coli* ATCC25922 and *S. aureus* ATCC25923 (Tables 1 and 5). Earlier studies have reported strong synergism between rifampicin and other antimicrobial agents (Timurkaynak et al. 2006). This makes rifampicin a strong candidate for combination antimicrobial therapies. Among all the strains, *E. coli* ATCC25922 was the most susceptible to extracts (methanolic, *n*-hexane and seed oil) in combination with four commercial antibiotics (rifampicin, ciprofloxacin, cefotaxime and ofloxacin). Among antibiotics, highest antagonistic effects were shown by moxifloxacin in combination with all extracts with the only exception of synergistic activities with methanolic extract against *A. baumannii* and *S. aureus* strains (Table 1). Aqueous extract was least active in combinatorial treatments and exhibited no synergistic activities against *E. coli*, *E. faecalis*, *P. vulgaris and S. aureus*. Aqueous extracts are known to exhibit relatively lower antibacterial activities compared to methanolic or *n*-hexane extract (Matu and Van Staden 2003). The present study found that extracts were more potent in combination than they were individually, against selected MDR strains. It is suggested that plant extracts hypothetically increase the efficacy of antibiotics against MDR strains and inhibit their efflux pumps or change resistance properties by releasing their antimicrobial compounds (Stermitz et al. 2000). In combination treatments, seed oil was the least active against MDRs and exhibited synergism only in combination with ofloxacin against MRSA1. The seed oil did not exhibit any combined effect against remaining MDR strains. The possible reason may be the instability of long chain fatty acids (Loftsson et al. 2016), or their tendency to bind non-specifically to proteins and other target compounds (Desbois and Smith 2010). Against MDR strains, moxifloxacin exhibited highest antagonism in combination with extracts and seed oil. However, seed oil exhibited higher synergism compared to the aqueous extract against clinical and reference strains. Literature has reported strong antimicrobial activity of oils (Thormar 2010).

The selected plant extracts and oil exhibited strong synergism with nucleic acids targeting antibiotics such as rifampicin, ofloxacin and ciprofloxacin. Rifampicin targets the DNA-dependent RNA polymerase inhibiting DNA-dependent RNA synthesis. Ofloxacin and ciprofloxacin mainly target DNA gyrase enzymes and inhibit bacterial cell division (Bébéar et al. 1998). There could be some possible reasons for this. Firstly, this strong synergism could be the result of interaction of the extracts and oil with outer membrane, cell wall and cell membrane and of antibiotics to nucleic acids. Firstly, this suggests that the extracts/oil target outer membranes allowing antibiotics to enter the cells, inhibiting nucleic acids machinery thus inhibiting cell division or apoptosis. Secondly, the two compounds used in combination form a new bioactive compound which has stronger antimicrobial effects (Vaara 1992). Thirdly, the phytochemicals present in *J. curcas* extracts may reduce inherited bacterial resistance. For instance, flavonoids and polyphenols; methanolic extract combined with antibiotics may have altered bacterial resistance thereby increasing combination treatment efficacy (Olajuyigbe and Afolayan 2012). Cefotaxime, a cell wall inhibitor, binds to penicillin-binding proteins inhibiting peptidoglycan synthesis. The present study has found relatively lower synergism exhibited by cefotaxime with extracts and oil. Moxifloxacin was an exception; although it is a broad-spectrum DNA gyrase inhibiting antibiotic but in the present study, it exhibited antagonism in most of the interactions. Further study is required to elucidate structural changes in compounds during interactions. Cefotaxime, a cell wall inhibitor, exhibited potent activities in combination with extracts and oil, indicating enhancement in its antibacterial potential. This claim is further strengthened by a study conducted by Zhao et al. (2001) where it is stated that cell wall targeting antibiotics exhibit increased activity when combined with phytochemicals targeting the same site. Moreover, efflux pump, an important tool for microbial resistance to antibiotics, is also affected by combination of antibiotics and phytochemicals (Coutinho et al. 2008). In view of the previous combination therapy studies, it is held that phytochemicals from *J. curcas* seed oil and de-oiled seed extracts combined with some antibiotics can make human pathogenic clinical bacterial and MDR strains more sensitive. Purification of these phytochemicals and their utilization in combination with commercially available antibiotics against pathogenic bacteria in nosocomial and other infections could prove to be the next step in the discovery of new antibiotics to combat antibiotic resistance in bacteria.

The combination of antimicrobial compounds showing in vitro synergistic activities against infectious agents are considered ideal options for effective treatment of bacterial infections, especially in patients with hardly curable infections. Since the discovery and development of new classes of potent antibiotics is the need of the day, the crude extracts and seed oil of *J. curcas* appear to be promising as these exhibited potent antibacterial activities against varied clinical pathogenic and multidrug resistant bacterial strains. Among all the de-oiled seed cake extracts and seed oil of *J. curcas*, crude methanolic extracts exhibited comparatively more potent antibacterial activities both individually as well as in combination with selected commercial antibiotics. In the current study, methanolic extracts were found with higher synergism compared to the *n*-hexane, aqueous extracts and seed oil in combination with commercially available antibiotics against selected strains. Especially, rifampicin had strong synergistic effects in combination with methanolic extract against various bacterial strains and is strongly recommended for combination therapies. The extracts and antibiotics combinations with higher synergism are suggested for effective therapy of infectious diseases caused by clinical and multi drug resistant pathogenic strains. Hence, evaluating the therapeutic potential of *J. curcas* allows one to see how it could be used best in combination with commercial antibiotics for effective treatment of bacterial diseases, especially, when the synergistic competency between plants and commercially available antibiotics is required for effective therapy. In addition, the utilization of *J. curcas* seeds for antimicrobial activities along with biofuels production in biorefinery concept may help to boost the economic viability of biofuel technology. This study has indicated the potential of *J. curcas* as a source of resistance modulation and novel chemotherapeutic agents for the production of synthetically improved therapeutic agents that can be used in combination with antibiotics, enhancing their antibacterial potential. However, further research is required to extract potential phytochemicals in pure form from *J. curcas* pressed seed cake and seed oil and to evaluate their effects on pathogenic microorganisms. In addition, it would also be interesting if the mechanism of action of these extracts, on target microorganisms, is determined individually as well as in combination with other drugs of choice that are unable to treat these resistant pathogenic microorganisms individually.

## Additional file

**Additional file 1.** Additional tables and figures.

## Data Availability

All relevant data are included in this manuscript.

## Abbreviations

DMSO: dimethyl sulfoxide
MHA: Mueller–Hinton agar
ATCC: American type culture collection
MDR: multidrug resistant
MRSA: methicillin-resistant *Staphylococcus aureus*
CFU: colony forming units
OD: optical density
GC–MS: gas chromatography coupled with mass spectrometry
FTIR: fourier transform infrared radiation
MIC: minimum inhibitory concentration
FIC: fractional inhibitory concentration
FICI: fractional inhibitory concentration index
MurF: UDP-*N*-acetylmuramoyl-tripeptide–d-alanyl-d-alanine
