# Supplementary material for: Comprehensive investigation on the synergistic antibacterial activities of Jatropha curcas pressed cake and seed oil in combination with antibiotics
Source: AMB Express. 2019 May 17;9:67. doi: 10.1186/s13568-019-0793-6 (PMC6525210; doi:10.1186/s13568-019-0793-6)
Supplement: Supplementary file 1 — Additional file 1. Additional tables and figures. [file 13568_2019_793_MOESM1_ESM.docx]

**Supplementary Data**

**Applied Microbiology and Biotechnology Express**

**Comprehensive Investigation on the Synergistic Antibacterial Activities of *Jatropha Curcas* Pressed Cake and Seed Oil in Combination with Antibiotics**

Abdul Haq^1^, Maleeha Siddiqi^1^, Syeda Zakia Batool^1^, Alam Khan^1^, Dildar Khan^2^, Samiullah Khan^1^, Arshad Islam^3,†^, Haji Khan^4^, Aamer Ali Shah^1^, Fariha Hasan^1^, Safia Ahmed and Malik Badshah^1,*^

^1^Department of Microbiology, Faculty of Biological Sciences, Quaid-i-Azam University, Islamabad 45320, Pakistan. E-mails: [haq_afridi88@yahoo.com](mailto:haq_afridi88@yahoo.com) (A.H.); [maleeha.s91@gmail.com](mailto:maleeha.s91@gmail.com) (M.S); [s.zak90@yahoo.com](mailto:s.zak90@yahoo.com) (S.Z.B); [khanalam0017@gmail.com](mailto:khanalam0017@gmail.com) (A.K); [samikhan@qau.edu.pk](mailto:samikhan@qau.edu.pk) (S.K); [alishah@qau.edu.pk](mailto:alishah_75@yahoo.com%3Cbr%3E%20alishah@qau.edu.pk) (A.A.S); [farihahasan@yahoo.com](mailto:farihahasan@yahoo.com) (F.H); [sahmed@qau.edu.pk](mailto:sahmed@qau.edu.pk) (S.A); [malikbadshah@gmail.com](mailto:malikbadshah@gmail.com) (M.B)

^2^Department of Pharmacy, Quaid-i-Azam University, Islamabad 45320, Pakistan. E-mail: [dildarafridi3@gmail.com](mailto:dildarafridi3@gmail.com) (D.K.)

^3^Postgraduate Program in Physiology and Pharmacology, Institute of Biological Sciences, Universidade Federal de Minas Gerais, Belo Horizonte-312.709.01, Minas Gerais, Brazil. Email: [arshad.cgl@gmail.com](mailto:arshad.cgl@gmail.com) (A.I.)

^4^Centre for Biotechnology and Microbiology, University of Swat, 19200, Pakistan. Email: [hkbiotech@gmail.com](mailto:hkbiotech@gmail.com) (H.K.)

^†^Present Address: Sulaiman Bin Abdullah Aba Al Khail Centre for Interdisciplinary Research in Basic Sciences, International Islamic University, Islamabad 44000, Pakistan

^*^Corresponding author: Dr. Malik Badshah. Email: [malikbadshah@gmail.com](mailto:malikbadshah@gmail.com), ORCID ID: [https://orcid.org/0000-0003-1913-954X](https://orcid.org/0000-0003-1913-954X). Tel: +92-51 9064-3255.


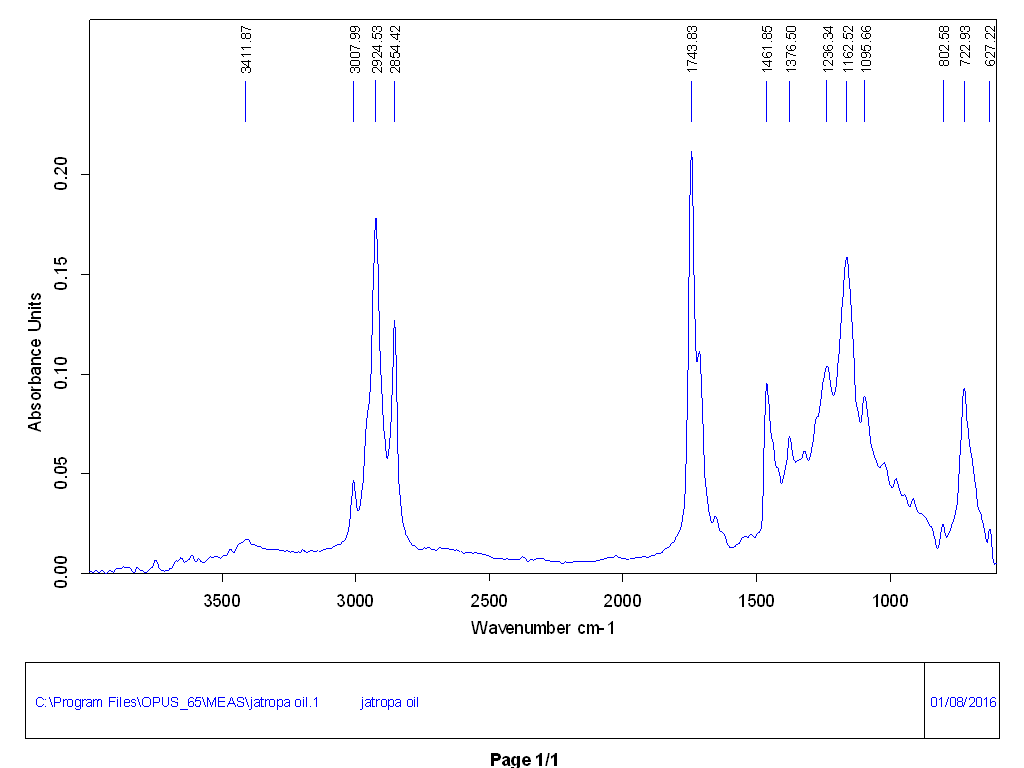


**Fig. S1** FTIR absorption spectrum obtained for *J. curcas* seed oil in the range of 4000-400 cm^-1^.

**
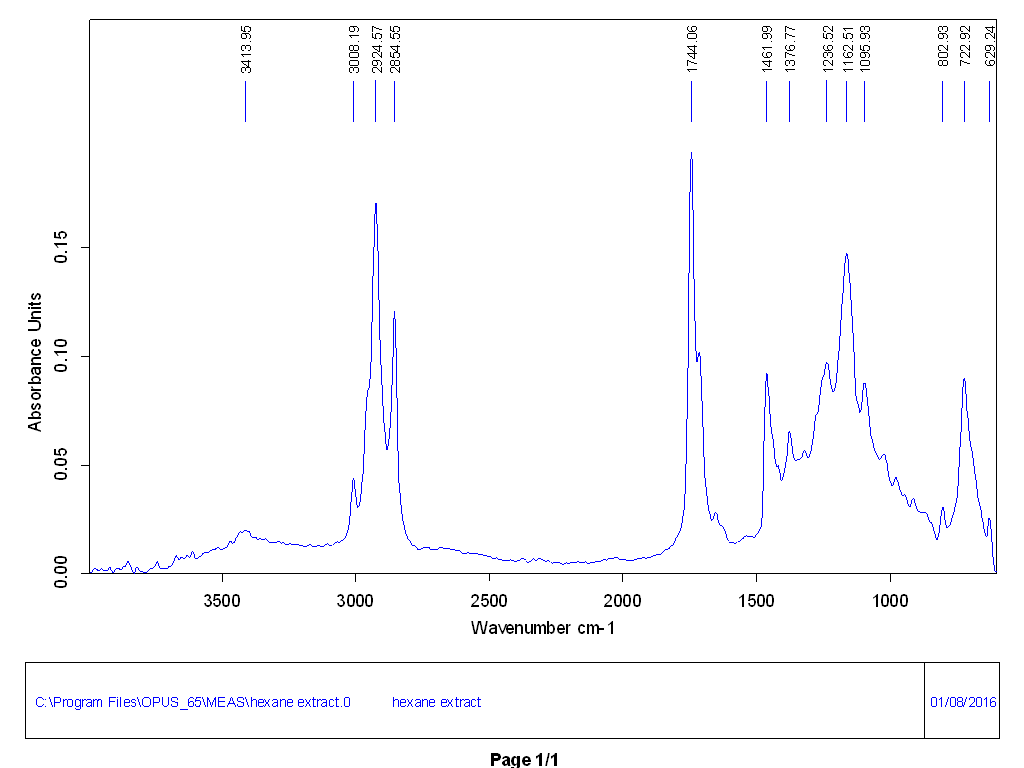
**

**Fig. S2** FTIR absorption spectrum obtained for n-hexane extract of *J. curcas* de-oiled seed in the range of 4000-400 cm^-1^.

**
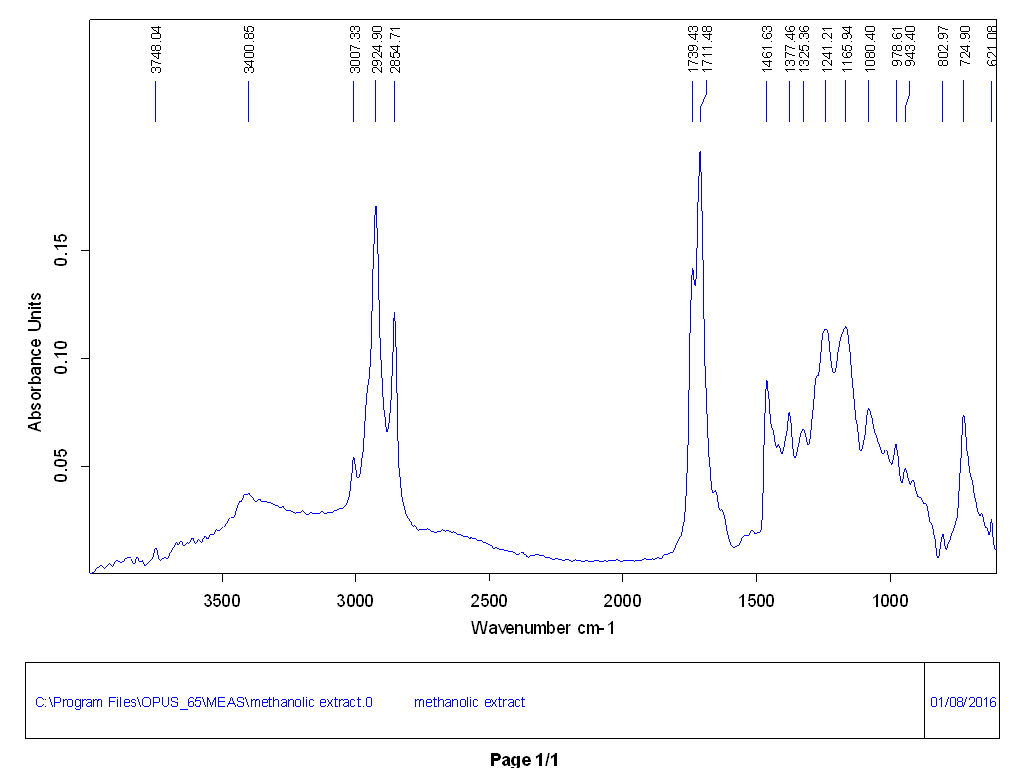
**

**Fig. S3** FTIR absorption spectrum obtained for methanolic extract of *J. curcas* de-oiled seed in the range of 4000-400 cm^-1^.

**
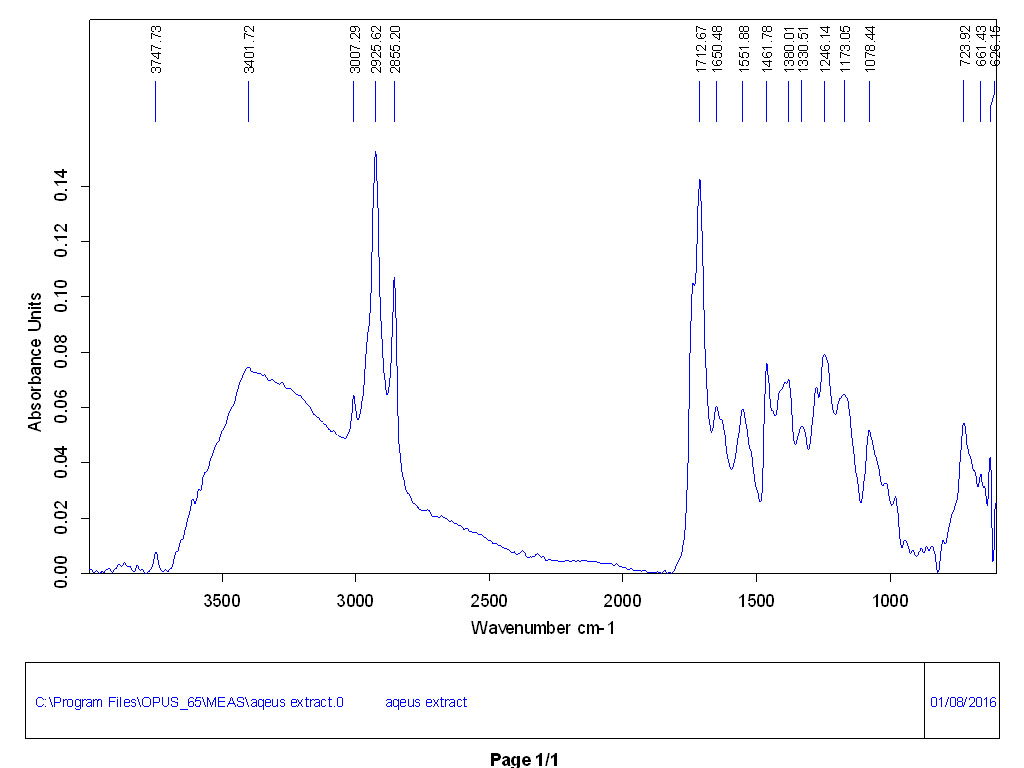
**

**Fig. S4** FTIR absorption spectrum obtained for aqueous extract of *J. curcas* de-oiled seed in the range of 4000-400 cm^-1^.

**
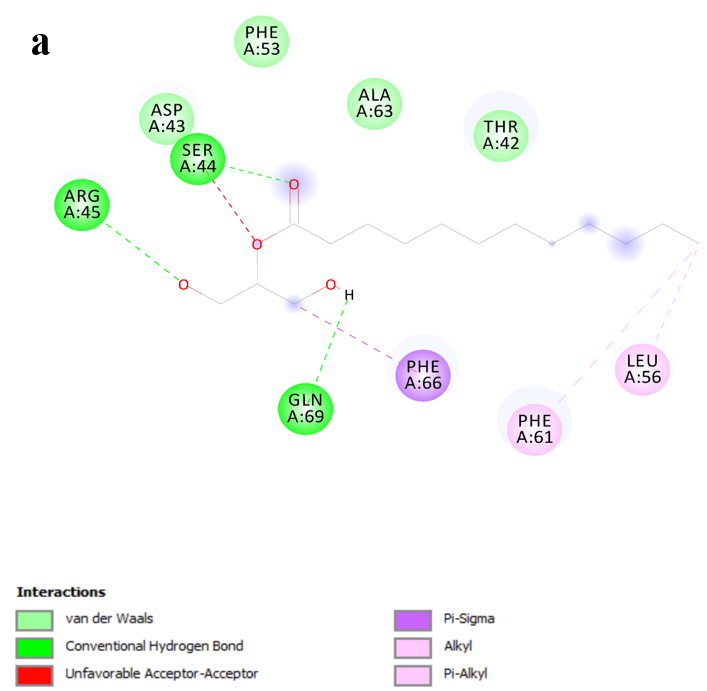
**


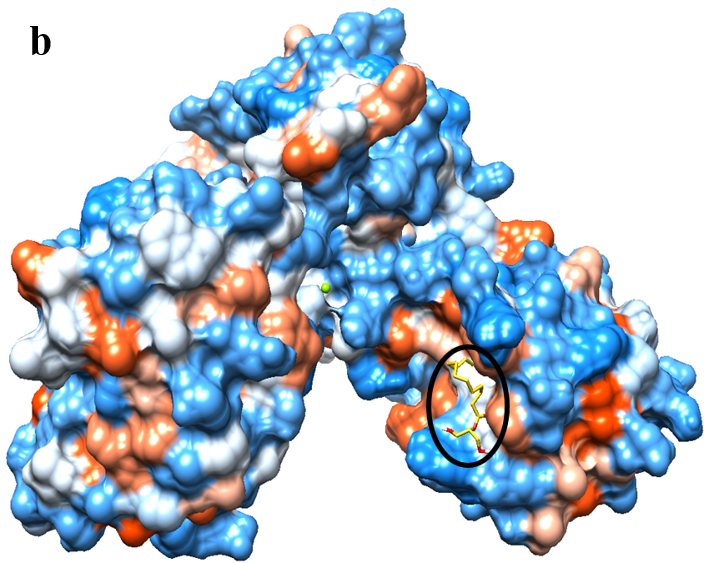


**Fig. S5** Molecular docking of beta-monolaurin in MurF active pocket **(a)** Binding interaction of beta-monolaurin in MurF active pocket, **(b)** Binding conformation of beta-monolaurin in MurF active pocket.

**
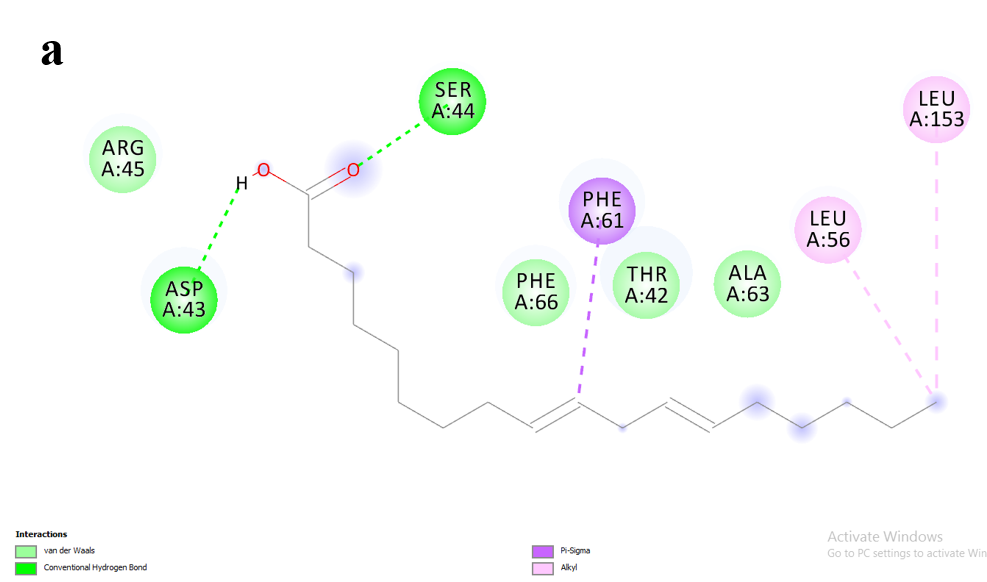

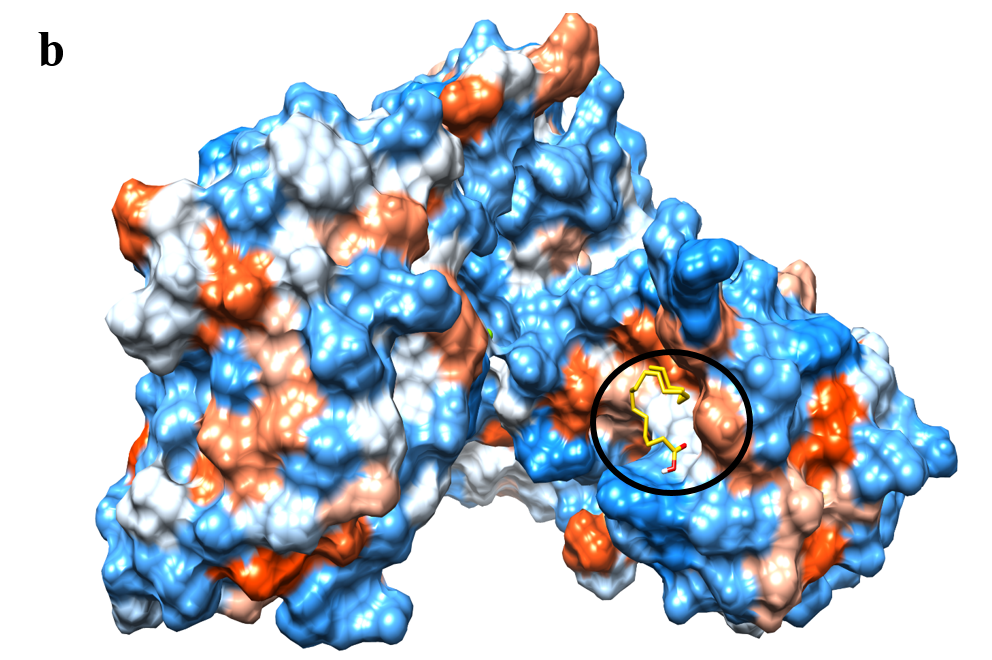
**

**Fig. S6** Molecular docking of 9,12 octadecadienoic acid in MurF active pocket **(a)** Binding interaction of 9,12 octadecadienoic acid in MurF active pocket, **(b)** Binding conformation of 9,12 octadecadienoic acid in MurF active pocket.

**Table S1** Antibiotic resistance profiling of MDR strains.

| **Bacterial strain** | **Antibiotics the strain is resistant to** |
| --- | --- |
| *K. pneumoniae* | Amikacin, Fucidic acid, Quinupristin-dalfopristin, Piperacillin-tazobactam, Vancomycin, Ceftriaxone, Oxacillin, Amoxicillin-clavulanate (2:1), Erythromycin, Cefoxitin, Meropenem, Cefazolin, Trimethoprim-sulfamethoxazole, |
| *P. chlororaphis* | Cefoxitin, Meropenem, Erythromycin, Nobycin, Amoxicillin-clavulanate (2:1), Cefazolin , Amikacin, Trimethoprim-sulfamethoxazole |
| *P. monteili* | Erythromycin, Cefoxitin, Trimethoprim-sulfamethoxazole, Meropenem, Tetracycline, Ampicillin, Nobycin, Tigecycline, Cefazolin, Amoxicillin-clavulanate (2:1) |
| *A. baumannii* | Ampicillin, Erythromycin, Trimethoprim-sulfamethoxazole, Amoxicillin-clavulanate (2:1), Ceftriaxone |
| *MRSA1* | Tetracyclin, Trimethoprime Sulfamethoxazole, Ciprofloxacin, Gentamicin |
| *MRSA2* | Tetracyclin, ciprofloxacin, Gentamicin, Erythromycin, Cefoxitin |
| *MRSA3* | Cefoxitin, Ciprofloxacin, Gentamicin |
| *MSSA* | Chloramphenicol, Tetracyclin, ciprofloxacin, |
| *MRSA5* | Tetracyclin, Cefoxitin, Ciprofloxacin, Clindamycin, Fosfomycin |

**Table S2** Assignments of the important FTIR absorption bands to corresponding functional groups present in *J. curcas* seed oil

| **No.** | **Band positions (cm^-1^)** | **Inferences of FTIR Spectrum** |
| --- | --- | --- |
| 1 | 1743 ^s^ | C=O Ester Stretch |
| 2 | 3411 ^s^ | carboxylic acid OH stretch, N-H stretch, alcohol OH stretch |
| 3 | 3007 ^w^ | =C-H stretch |
| 4 | 2924 ^w^ | -C-H stretch |
| 5 | 2854 ^w^ | -C-H stretch |
| 6 | 1461 ^m^ | CH_3_ , CH_2_ stretches |
| 7 | 1376 ^m^ | CH_3_ stretches |
| 8 | 1236 ^s^ | C-O-C stretch |
| 9 | 1162, 1095 ^s^ | C-OH stretch |

^s^: strong; ^w^: weak; ^m^: medium

**Table S3** FTIR stretches with corresponding functional groups present in n-hexane extract of *J. curcas* seed

| **No** | **Band positions (cm^-1^)** | **Inferences of FTIR Spectrum** |
| --- | --- | --- |
| **1** | 1744 ^s^ | C=O Ester Stretch |
| **2** | 3413 ^s^ | carboxylic acid OH stretch, N-H stretch, alcohol OH stretch |
| **3** | 3008 ^w^ | =C-H stretch |
| **4** | 2924 ^w^ | -C-H stretch |
| **5** | 2854 ^w^ | -C-H stretch |
| **6** | 1461 ^m^ | CH_3_ , CH_2_ stretches |
| **7** | 1376 ^s^ | CH_3_ stretches |
| **8** | 1236 ^s^ | C-O-C stretch |
| **9** | 1162, 1096 ^s^ | C-OH stretch |
| **10** | 802, 722, 629 ^s^ | CH out of plane bending (carbohydrate) |

^s^: strong; ^w^: weak; ^m^: medium

**Table S4** FTIR stretches with corresponding functional groups present in methanolic extract of *J. curcas* seed

| **No** | **Band positions (cm-1)** | **Inferences of FTIR Spectrum** |
| --- | --- | --- |
| **1** | 1744 ^s^ | C=O Ester Stretch |
| **2** | 3413 ^s^ | carboxylic acid OH stretch, N-H stretch, alcohol OH stretch |
| **3** | 3008 ^w^ | =C-H stretch |
| **4** | 2924 ^w^ | -C-H stretch |
| **5** | 2854 ^w^ | -C-H stretch |
| **6** | 1461 ^m^ | CH_3_ , CH_2_ stretches |
| **7** | 1376 ^m^ | CH_3_ stretches |
| **8** | 1236 ^s^ | C-O-C stretch |
| **9** | 1162, 1096 ^s^ | C-OH stretch |
| **10** | 802, 724, 621 ^s^ | CH out of plane bending (carbohydrate) |

^s^: strong; ^w^: weak; ^m^: medium

**Table S5** FTIR stretches with corresponding functional groups present in aqueous extract of *J. curcas* seed

| **No** | **Band positions (cm-1)** | **Inferences of FTIR Spectrum** |
| --- | --- | --- |
| **1** | 1712 ^s^ | C=O ketone Stretch |
| **2** | 3401 ^s^ | carboxylic acid OH stretch, N-H stretch, alcohol OH stretch |
| **3** | 3007 ^w^ | =C-H stretch |
| **4** | 2925 ^w^ | -C-H stretch |
| **5** | 2855 ^w^ | -C-H stretch |
| **6** | 1461 ^m^ | CH_3_ , CH_2_ stretches |
| **7** | 1380 ^m, s^ | CH_3_ stretches, NO_2_stretch |
| **8** | 1246 ^s^ | C-O-C stretch |
| **9** | 1173, 1078 ^s^ | C-OH stretch |
| **10** | 723, 661, 626 ^s^ | CH out of plane bending (carbohydrate) |
| **11** | 1650 ^w^ | C=C alkene stretches |
| **12** | 1551 ^s^ | C=O amide, C=C aromatic stretches |
| **13** | 1330 ^s^ | NO_2_stretch |

^s^: strong; ^w^: weak; ^m^: medium

**Table S6** Types and structures of fatty acids present in *J. curcas* oil identified by GC-MS analysis

| **Compound Name** | **Molecular structure** | **MW** | **RT** | **Area%** | **Height%** |
| --- | --- | --- | --- | --- | --- |
| Octanoic acid |  | 144 | 8.90 | 0.24 | 0.77 |
| Nonanoic acid |  | 158 | 8.90 | 0.24 | 0.77 |
| n-Decanoic acid |  | 172 | 11.95 | 0.25 | 1.23 |
| Dodecanoic acid |  | 200 | 14.63 | 1.59 | 5.23 |
| Undecanoic acid |  | 186 | 14.63 | 1.59 | 5.23 |
| Tridecanoic acid |  | 214 | 14.63 | 1.59 | 5.23 |
| Myristic acid |  | 228 | 16.95 | 3.54 | 9.51 |
| Tridecanoic acid |  | 214 | 16.95 | 3.54 | 9.51 |
| n-Hexadecanoic acid |  | 256 | 19.083 | 6.62 | 10.35 |
| Pentadecanoic acid |  | 242 | 19.083 | 6.62 | 10.35 |
| Arachidic acid |  | 312 | 19.083 | 6.62 | 10.35 |
| Palmitic acid |  | 256 | 19.083 | 6.62 | 10.35 |
| Tetradecanoic acid |  | 228 | 19.083 | 6.62 | 10.35 |
| Stearic acid |  | 284 | 21.15 | 51.60 | 24.39 |
| 11-Eicosenoic acid |  | 324 | 22.67 | 17.08 | 19.45 |
| Erucic acid |  | 338 | 24.33 | 12.58 | 19.46 |

^M.W.: molecular weight; RT: Retention time^

**Table S7** Structures of bioactive compounds present in *J. curcas* n-hexane extract identified by GC-MS analysis

| **Compound Name** | **Molecular structure** | **MW** | **RT** | **Area%** | **Height%** |
| --- | --- | --- | --- | --- | --- |
| Oleic acid |  | 282 | 27 | 5.72 | 4.24 |
| 9,12-Octadecadienoic acid (Z,Z)- |  | 280 | 25.33 | 70.61 | 61.37 |
| Palmitic acid |  | 256 | 23.54 | 9.28 | 14.89 |
| Myristic acid |  | 228 | 21.47 | 5.08 | 9.19 |
| Di-n-octyl phthalate |  | 390 | 28.75 | 2.33 | 4.67 |

^M.W.: molecular weight; RT: Retention time^

**Table S8** Structures of bioactive compounds present in *J. curcas* methanolic extract identified by GC-MS analysis

| **Compound Name** | **Molecular structure** | **MW** | **RT** | **Area%** | **Height%** |
| --- | --- | --- | --- | --- | --- |
| Diacetone alcohol |  | 116 | 4.892 | 38.42 | 5.52 |
| I-(+)-Ascorbic acid 2,6-dihexadecanoate |  | 652 | 23.51 | 0.19 | 0.15 |
| 9-hexadecenal |  | 238 | 25.25 | 4.95 | 5.06 |
| Beta-Monolaurin |  | 274 | 28.53 | 0.27 | 0.32 |
| bis (tridecyl) phthalate |  | 530 | 28.75 | 1.61 | 4.78 |
| 1-docosanol |  | 326 | 29.35 | 1.20 | 1.15 |

^M.W.: molecular weight; RT: Retention time^

**Table S9** Structures of bioactive compounds present in *J. curcas* aqueous extract identified by GC-MS analysis

| **Compound Name** | **Molecular structure** | **MW** | **RT** | **Area%** | **Height%** |
| --- | --- | --- | --- | --- | --- |
| 1,4-Dithiane |  | 120 | 14.53 | 2.95 | 4.38 |
| Dodecanoic acid methyl ester |  | 214 | 18.51 | 0.51 | 0.50 |
| Methyl Tetradecanoate |  | 242 | 20.90 | 1.42 | 0.19 |
| Vitamin D3 |  | 384 | 21.45 | 1.89 | 3.12 |
| Palmitic acid, methyl ester |  | 270 | 23.03 | 1.07 | 3.16 |
| Palmitic acid |  | 256 | 23.52 | 2.96 | 5.11 |
| Isopropyl linoleate |  | 322 | 24.76 |  |  |
| Di-n-octyl phthalate |  | 390 | 28.73 | 1.61 | 4.79 |

^M.W: molecular weight; RT: Retention time^

**Table S10** Binding energy of the structures present in *J. curcas* methanolic extract found by using molecular docking

| **Compound Name** | **Molecular structure** | **Binding energy (kcal/mol)** |
| --- | --- | --- |
| Diacetone alcohol |  | -4.5 |
| I-(+)-Ascorbic acid 2,6-dihexadecanoate |  | -2.3 |
| 9-hexadecenal |  | 5.5 |
| Beta-Monolaurin |  | -7.3 |
| Bis-(tridecyl) phthalate |  | -6.8 |
| 1-docosanol |  | -1.1 |

**Table S11** Binding energy of the structures present in *J. curcas* n-hexane extract found by using molecular docking

| **Compound Name** | **Molecular structure** | **Binding energy (kcal/mol)** |
| --- | --- | --- |
| Oleic acid |  | -2.1 |
| 9,12-Octadecadienoic acid (Z,Z)- |  | -6.2 |
| Palmitic acid |  | -4.1 |
| Myristic acid |  | -1.2 |
| Di-n-octyl phthalate |  | -5.1 |
